# Shared origins of a key enzyme during the evolution of C_4_ and CAM metabolism

**DOI:** 10.1093/jxb/eru087

**Published:** 2014-03-17

**Authors:** Pascal-Antoine Christin, Monica Arakaki, Colin P. Osborne, Andrea Bräutigam, Rowan F. Sage, Julian M. Hibberd, Steven Kelly, Sarah Covshoff, Gane Ka-Shu Wong, Lillian Hancock, Erika J. Edwards

**Affiliations:** ^1^Department of Animal and Plant Sciences, University of Sheffield, Western Bank, Sheffield S10 2TN, UK; ^2^Department of Ecology and Evolutionary Biology, Brown University, 80 Waterman St., Providence, RI 02912, USA; ^3^Departamento de Botánica, Facultad de Ciencias Biológicas and Museo de Historia Natural – UNMSM, Av. Arenales 1256, Lima 11, Peru; ^4^Institute of Plant Biochemistry, Heinrich-Heine University, 40225 Duesseldorf, Germany; ^5^Department of Ecology and Evolutionary Biology, University of Toronto, 25 Willcocks Street, Toronto, Ontario M5S 3B2, Canada; ^6^Department of Plant Sciences, University of Cambridge, Cambridge CB2 3EA, UK; ^7^Department of Plant Sciences, University of Oxford, Oxford OX1 3RB, UK; ^8^Department of Biological Sciences, University of Alberta, Edmonton, Alberta T6G 2E9, Canada; ^9^Department of Medicine, University of Alberta, Edmonton, Alberta T6G 2E1, Canada; ^10^BGI-Shenzhen, Beishan Industrial Zone, Yantian District, Shenzhen 518083, China

**Keywords:** C4 photosynthesis, CAM photosynthesis, co-option, evolution, phosphoenolpyruvate carboxylase (PEPC), phylogenetics.

## Abstract

CAM and C_4_ photosynthesis are two key plant adaptations that have evolved independently multiple times, and are especially prevalent in particular groups of plants, including the Caryophyllales. We investigate the origin of photosynthetic PEPC, a key enzyme of both the CAM and C_4_ pathways. We combine phylogenetic analyses of genes encoding PEPC with analyses of RNA sequence data of *Portulaca*, the only plants known to perform both CAM and C_4_ photosynthesis. Three distinct gene lineages encoding PEPC exist in eudicots (namely *ppc-1E1*, *ppc-1E2* and *ppc-2*), one of which (*ppc-1E1*) was recurrently recruited for use in both CAM and C_4_ photosynthesis within the Caryophyllales. This gene is present in multiple copies in the cacti and relatives, including *Portulaca*. The PEPC involved in the CAM and C_4_ cycles of *Portulaca* are encoded by closely related yet distinct genes. The CAM-specific gene is similar to genes from related CAM taxa, suggesting that CAM has evolved before C_4_ in these species. The similar origin of PEPC and other genes involved in the CAM and C_4_ cycles highlights the shared early steps of evolutionary trajectories towards CAM and C_4_, which probably diverged irreversibly only during the optimization of CAM and C_4_ phenotypes.

## Summary

Using phylogenetics and transcriptomics, we show that independent origins of both CAM and C_4_ photosynthesis in Caryophyllales co-opted the same genes for PEPC through similar adaptive changes.

## Introduction

During the evolutionary diversification of organisms, ecological selection pressures sometimes lead to the emergence of similar phenotypes in distantly related species. A good example of such convergent evolution is the recurrent emergence of CO_2_-concentrating mechanisms (CCMs) as an adaptation to environmental CO_2_ depletion (Raven *et al.*, 2008). CCMs have arisen through the assembly of novel biochemical pathways, which increase the internal concentration of CO_2_ around Rubisco before its fixation by the C_3_ photosynthetic cycle (Christin and Osborne, 2013). In flowering plants, the most frequent and successful CCMs are C_4_ and CAM photosynthesis (Keeley and Rundel, 2003). The C_4_ and CAM CCMs differ in the overall mechanism of atmospheric CO_2_ concentration, but the biochemical cycles are similar (Osmond, 1978; Hatch, 1987). Plants of both types fix inorganic carbon by phosphoenolpyruvate carboxylase (PEPC). In C_4_ plants, the resulting acid is typically modified and transported to another cell, where CO_2_ is released to feed the C_3_ cycle that is active within these cells (Hatch, 1987; Sage *et al.*, 2012). In CAM plants, a similar CCM occurs in which the initial carboxylation by PEPC and subsequent decarboxylation and refixation by Rubisco are temporally rather than physically separated. At night, PEPC fixes inorganic carbon into organic compounds, which are stored as malate in the vacuole until daytime, when the cycle is completed and CO_2_ is released to supply the C_3_ cycle (Osmond, 1978; Borland and Taybi, 2004).

The CAM and C_4_ pathways are complex traits, involving dozens of enzymes that fulfil different functions compared with the isoforms in the C_3_ ancestors (Bräutigam *et al.*, 2011; Gowik *et al.*, 2011). For the constituent enzymes investigated so far, the new function requires alterations in the expression pattern and/or its catalytic properties (Finnegan *et al.*, 1999; Tausta *et al.*, 2002; Svensson *et al.*, 2003; Gowik and Westhoff, 2011; Ludwig, 2011). For instance, the PEPC enzyme is ubiquitous in plants, where multiple isoforms are responsible for various non-photosynthetic functions (Lepiniec *et al.*, 1994; Aubry *et al.*, 2011; Gowik and Westhoff, 2011). The C_4_-specific forms evolved from non-photosynthetic genes through adaptation of the catalytic properties to the new metabolic context (Dong *et al.*, 1998; Gowik and Westhoff, 2011). In particular, the affinity of PEPC for its substrate PEP was decreased, and its sensitivity to feedback inhibition by malate was reduced (Bauwe and Chollet, 1986; Bläsing *et al.*, 2000; Svensson *et al.*, 2003; Gowik *et al.*, 2006; Lara *et al.*, 2006). In C_4_ grasses and sedges, this was achieved through numerous adaptive amino acid changes (Christin *et al.*, 2007; Besnard *et al.*, 2009).

Both the CAM and C_4_ pathways also require a specialized leaf anatomy (Hattersley, 1984; Nelson *et al.*, 2005). Despite this apparent complexity, the C_4_ trait evolved a minimum of 62 times independently in flowering plants (Sage *et al.*, 2011). While a precise tally is not yet available, it is likely that the total number of CAM origins will be even greater (Edwards and Ogburn, 2012). These numerous origins of novel photosynthetic types are, however, not evenly distributed across the phylogeny of flowering plants. While certain major lineages completely lack CCMs, others present a large number of independent origins of CAM, C_4_ or both (Crayn *et al.*, 2004; Sage *et al.*, 2011; Edwards and Ogburn, 2012). The high occurrence of C_4_ origins in some groups of angiosperms has been explained by different factors, with an emphasis on ecological and anatomical predispositions, as well as possible genomic predispositions (Sage, 2001; Monson, 2003; Christin *et al.*, 2013a, 2013b; Griffiths *et al.*, 2013). The existence of anatomical and/or ecological predispositions for CAM evolution might similarly explain the repeated incidence of this CCM in some groups (Edwards and Ogburn, 2012), although this has never been rigorously tested.

The frequency of occurrence of CCMs is particularly high in the eudicot clade Caryophyllales, which encompasses many C_4_ origins (Kadereit *et al.*, 2003; Christin *et al.*, 2011; Sage *et al.*, 2011; Kadereit *et al.*, 2012) and also several CAM lineages, including constitutive CAM (e.g. cacti) and facultative CAM types that can switch to CAM depending on environmental conditions (Guralnick and Ting, 1987; Guralnick *et al.*, 2008; Nyffeler *et al.*, 2008). Of special interest are species of *Portulaca*, which are the only plants known to be capable of performing both C_4_ and CAM cycles (Koch and Kennedy, 1980, 1982; Guralnick *et al.*, 2002). The majority of *Portulaca* species are C_4_ plants, with the associated anatomical specialization, but several species exhibit CAM-like physiology when grown in water-limited conditions (Kraybill and Martin, 1996; Guralnick *et al.*, 2002). Exposure to drought triggers physiological and biochemical changes in these *Portulaca* species, with different expression levels and catalytic properties of several C_4_/CAM enzymes, and slight alterations in their leaf anatomy (Mazen, 1996, 2000; Lara *et al.*, 2003, 2004). In molecular phylogenies, *Portulaca* is nested within Portulacineae (Nyffeler and Eggli, 2010; Ocampo and Columbus, 2010; Arakaki *et al.*, 2011), and is apparently the only C_4_ member of this group that is otherwise rich in species with varying degrees of CAM photosynthesis, the best known of which are cacti. Despite these intriguing patterns, neither sequence nor expression data for known C_4_/CAM genes have been analysed in *Portulaca* or its relatives.

The large number of CCM origins within Caryophyllales might suggest that this clade is especially prone to transitions between photosynthetic types. However, the history of photosynthetic transitions within the clade is still unclear. In particular, it is unknown if CAM and C_4_ origins represent completely independent evolutionary phenomena, or are distinct end-points to a partially shared evolutionary trajectory (Edwards and Ogburn, 2012). To increase our understanding of CCM origins in Caryophyllales, we studied the evolution of genes encoding PEPC, a key enzyme common to all CAM and C_4_ cycles. In addition, we investigated in detail the transcriptome of *Portulaca* individuals operating either the C_4_ or CAM cycles. Our comparative analyses shed new light on the shared history of genes involved in CAM and C_4_ photosynthesis.

## Material and methods

### Diversity of genes encoding PEPC in plants

To reconstruct the history of the multigene family encoding PEPC, complete cDNA sequences available in the GenBank database were first retrieved. These were used as a query of BLAST searches against completely sequenced nuclear genomes available on Phytozome (Goodstein *et al.*, 2012). This initial dataset was increased by screening genomic DNA from representatives of diverse Caryophyllales lineages and photosynthetic types through PCR to isolate genes encoding PEPC (Supplementary Tables S1 and S2, available at *JXB* online). These DNAs were first screened with primers *ppc-1204-For* and *ppc-2890-Rev* previously used to isolate PEPC genes from Molluginaceae, a family from Caryophyllales (Christin *et al.*, 2011). These primers amplify a fragment encompassing exons 8 to 10, which represents about half of the whole coding sequence and is known to include major determinants of the C_4_-specific properties of PEPC (Bläsing *et al.*, 2000; Engelmann *et al.*, 2002). PCR, cloning and sequencing were performed as previously described (Christin *et al.*, 2011). Preliminary analyses indicated that numerous PEPC genes were present in some Caryophyllales genomes. To increase the likelihood of sampling specific copies, additional primers were designed with the purpose of increasing the PCR specificity for certain gene lineages (Supplementary Table S3, available at *JXB* online). These additional PCRs were conducted as for the primers *ppc-1204-For* and *ppc-2890-Rev*. The PCR products were purified and directly sequenced with one of the primers used for the PCR. Chromatograms were visually inspected and PCR products were cloned only if multiple genes were detected by the presence of overlapping peaks.

**Fig. 1.**
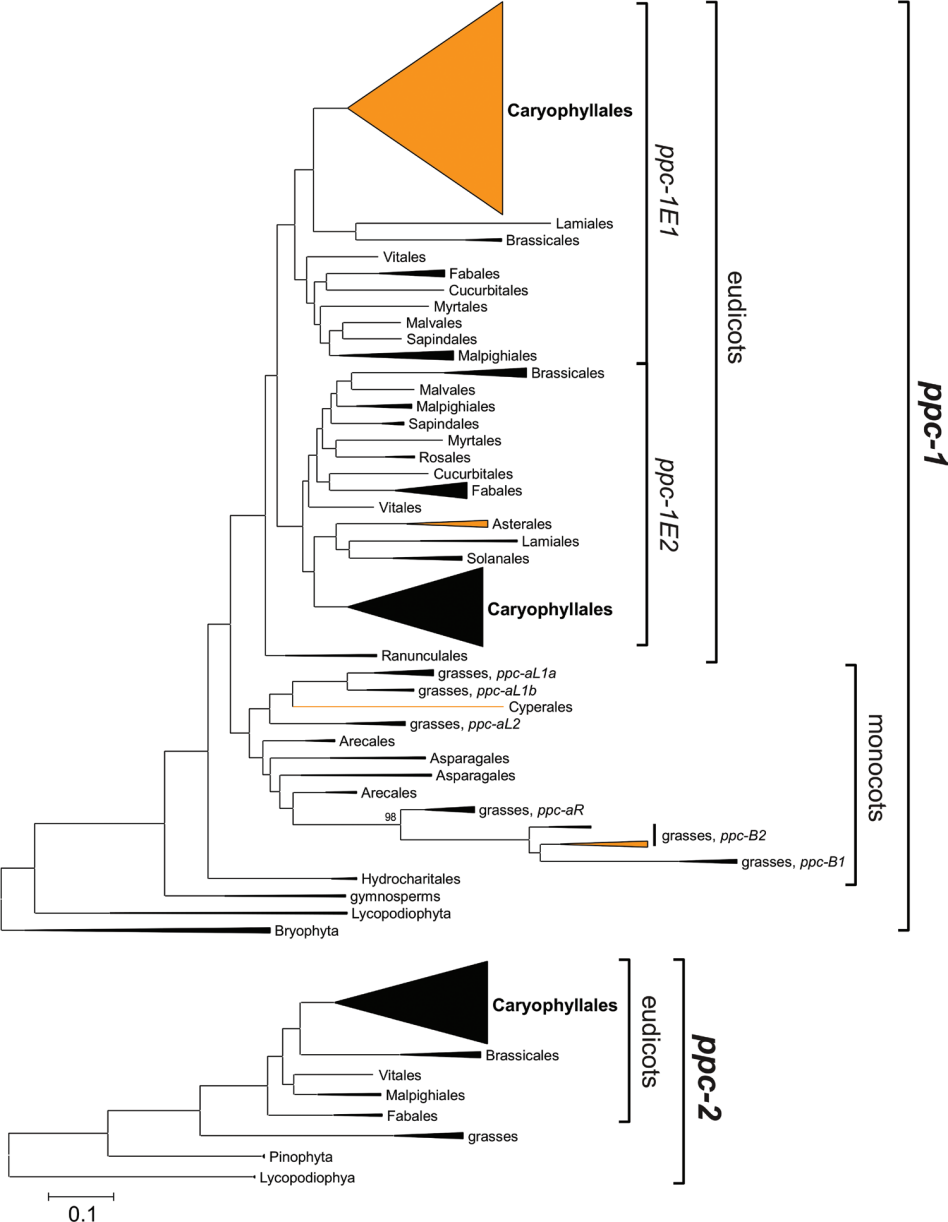
Phylogenetic relationships among PEPC-encoding genes from land plants. The phylogenetic trees were obtained through Bayesian inference on each main gene lineage; *ppc-1* and *ppc-2*. Taxonomic groups are compressed, with the size of triangles proportional to the number of sequences in the group. Gene lineages and main clades of flowering plants are delimited on the right. Clades containing some genes with a Ser780 are in orange. Details, including support values, are available in Supplementary Figs S1 and S2 (available at *JXB* online). The scale bar represents expected substitutions per site. (This figure is available in colour at *JXB* online.)

Exons were identified by homology to annotated sequences and following the GT-AG rule. All coding sequences, retrieved from public databases or isolated through PCR, were translated into protein sequences and aligned with ClustalW (Thompson *et al.*, 1994). The alignment was visually inspected, manually refined, and replaced with the corresponding nucleotide sequences, which were later used for analyses. A preliminary phylogenetic tree identified two distantly related groups of PEPC encoding genes that diverged before the evolution of land plants (*ppc-1* and *ppc-2*; Fig. 1), each represented by ferns, gymnosperms, and flowering plants. Despite clear homology, the two groups were highly divergent, leading to ambiguities in the alignment. Each group was consequently analysed separately.

### Transcriptome analysis of *Portulaca oleracea*

The species *Portulaca oleracea* was selected for transcriptome analyses to identify CAM- and C_4_-specific genes, with a special focus on PEPC. Seeds originating from Syria were provided by the USDA-ARS (GRIN accession: Grif 14515). Plants were grown in 3-inch pots of equal-parts gravel/calcinated clay perlite mix, and in a Conviron E7/2 plant growth chamber (Conviron Ltd., Winnipeg, Manitoba, Canada) with 14 hours of daylight. The growth chamber was illuminated with twelve 32-W fluorescent lamps and four 60-W incandescent lamps. Temperature was kept at 22°C from 3h before dark until after 3h of light, and was increased to 28°C for the middle portion of the light period. The position of pots within the growth chamber was randomized daily.

On the first day of the experiment (5 March 2012), all plants were bottom-watered and seedlings were split into two groups. One group was bottom-watered every 2–4 days, while the other group was bottom-watered less than once a week (Supplementary Table S4, available at *JXB* online). Nutrients were added to the water periodically at a concentration of 1:100 (w/v) of K:P:N=20:20:20. After 1 month under these conditions, leaves were collected, flash-frozen in liquid nitrogen, and stored at –80°C. Two individuals per group were sampled twice, after 4h of light and after 2h of dark. To control for stress effects that might have been induced by leaf cutting, the first individual from each group was sampled first during the day and then the following night, while the second individual was sampled first during the night and then the following day. An equivalent number of young and mature leaves were collected from the light and dark samples. Two additional individuals per group (watered frequently and watered occasionally) were sampled for acid titration (see below). One individual of each group was sampled first at the end of the dark period (1h before light) and then at the end of the light cycle (2h before dark). The sampling order was inverted for the second individual of the same group.

RNA was extracted from several leaf fragments using the FastRNA™ Pro Green Kit (MP Biomedicals, OH, USA). Several RNA extractions per sample (one individual in one condition) were pooled and prepared for sequencing using the Illumina TruSeq mRNA Sample Prep Kit (Illumina Inc., San Diego, CA, USA). Fragments of the cDNA libraries between 400 and 450bp long were selected, and the different samples were marked with specific bar-codes, multiplexed and sequenced using an Illumina HiSeq 2000 instrument, as paired-end 100bp reads.

### Titratable acidity

**Fig. 2.**
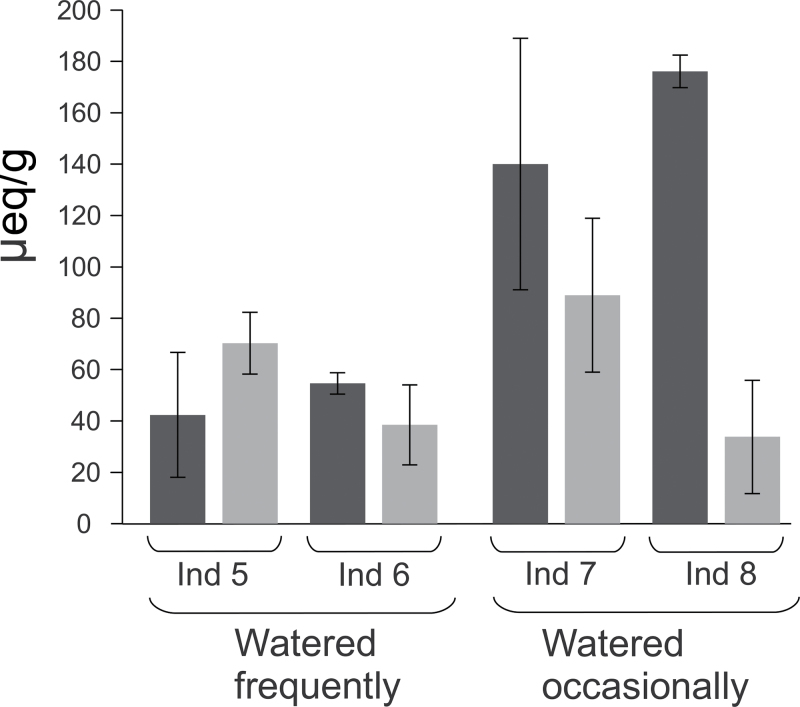
Effect of the water regime on titratable acidity. The titratable acidity (in microequivalents per gram of frozen weight, μeq/g) is indicated for samples taken at the end of the dark phase (dark grey) and at the end of the light phase (light grey). The error bars indicate standard deviations over three replicates.

The titratable acidity was measured at the end of the dark phase and at the end of the light phase. An accumulation of acids during the night followed by consumption during the day is indicative of a CAM cycle (Silvera *et al.*, 2005). Three leaves were analysed for each of the eight combinations of individuals/sampling time. Frozen leaves were weighed and immediately ground briefly in mortar and pestle and then boiled in 50ml 20% ethanol. After cooling, pH was slowly brought to 7 by adding 0.1 N NaOH in 5 or 10μl increments. The titratable acidity was calculated as the amount of H^+^ equivalents required to neutralize the leaf extracts (Silvera *et al.*, 2005). Despite variability among individuals and leaves, the plants watered occasionally showed a clear increase of titratable acidity at the end of the dark period (Fig. 2). These results confirm that less frequent watering triggered a CAM physiology in *Portulaca oleracea*.

### Illumina data assembly and phylogenetic annotation

The reads corresponding to each of the eight different samples were assembled individually using the software Trinity (Grabherr *et al.*, 2011) as implemented in the Agalma pipeline (Dunn *et al.*, 2013). Reads from each Illumina run were mapped against the corresponding individual assembly using the software Bowtie 2 (Langmead and Salzberg, 2012) and its mixed model, which allows unpaired alignments when paired alignments fail. Only one of the best alignments chosen randomly was reported per read. The number of reads aligned to each contig was used to compute reads per million of alignable reads (rpm). Sequencing and mapping statistics are given in Supplementary Table S5 (available at *JXB* online).

The relative transcript abundance of each gene within each of the C_4_-related multigene families was estimated by assigning contigs to gene lineages based on phylogenetic analyses (Christin *et al.*, 2013a). For genes encoding C_4_-related enzymes (Supplementary Table S6, available at *JXB* online), sequences available in Genbank for Caryophyllales and other plant lineages were extracted based on their annotation. A BLAST search was used to extract homologous loci for predicted cDNA from several completely sequenced genomes (*Arabidopsis thaliana*, *Brachypodium distachyon*, *Carica papaya*, *Glycine max*, *Oryza sativa*, *Populus trichocarpa*, *Sellaginella moellendorffii*, *Sorghum bicolor*, *Selaginella moellendorffii* and *Vitis vinifera*). For each C_4_-related enzyme, the dataset assembled from sequences retrieved from Genbank and complete genomes was used as a query of a BLAST search against each of the assembled transcriptomes. The longest matching region for each contig was extracted from the BLAST results when larger than 50bp. Each of these was successively aligned to the reference dataset using Muscle (Edgar, 2004). The resulting alignment was used to infer one phylogenetic tree per contig under maximum likelihood as implemented in PhyML (Guindon and Gascuel, 2003) using a GTR+G model. The resulting phylogenetic trees were inspected and each contig was assigned to a gene lineage if clearly nested within. The rpm values of all contigs assigned to a given gene lineage were summed to obtain an estimate of the transcript abundance of the gene lineage.

The assignment of contigs to gene lineages was repeated with a reference dataset composed of exons 8–10 of genes encoding PEPC. Caryophyllales sequences isolated by PCR or retrieved from Genbank were used if complete for the studied fragment and assigned to the gene lineage *ppc-1E1* based on phylogenetic analyses (see Results). In order to increase the size of this dataset, the corresponding segment of matching contigs from *Portulaca* transcriptomes that represented at least half of the studied fragment were manually aligned with the dataset. Three contigs that covered the whole studied segment and which were clearly different from each other, as well as from *Portulaca* genes isolated by PCR, were added to the reference dataset. All matching *Portulaca* contigs were then successively placed in a phylogeny with this reference dataset and the total rpm values of each *ppc-1E1* gene lineage was computed as described above.

### Screening of *ppc* genes from other *Portulaca* transcriptomes

The 1KP project has sequenced transcriptomes from 1000 different species of plants, including several *Portulaca* species (http://www.onekp.com/; Johnson *et al.*, 2012). RNA for *Portulaca* species was extracted from leaves sampled during the day. Of these, *P*. *cryptopetala* is a putative C_3_–C_4_ intermediate species (Voznesenskaya *et al.*, 2010). Three additional clades of *Portulaca* are represented in the 1KP project (clades based on Ocampo and Columbus, 2012 and Ocampo *et al.*, 2013); ‘Oleracea’ (*P*. *molokiniensis*, *P*. *oleracea*, *P*. *suffruticosa*), ‘Pilosa’ (*P*. *grandiflora, P*. *amilis*), and ‘Umbraticola’ (*P*. *umbraticola*). Sequences of the different *Portulaca* species corresponding to *ppc-1E1* were retrieved through a BLAST search with *ppc-1E1* isolated in the present study used as a query. Sequences were considered further only if they aligned with the reference dataset along more than 500bp. The selected sequences were aligned with the *ppc-1* sequences isolated from genomic DNA or extracted from the *P*. *oleracea* transcriptome generated in this study (see above). The alignment was manually refined and only the longest of groups of identical sequences from the 1KP data was used for analyses.

### Phylogenetic analyses and codon models

The two datasets composed of all *ppc-1* and *ppc-2* genes retrieved from available databases or generated in this study were used to infer phylogenetic trees with MrBayes 3.2.1 (Ronquist and Huelsenbeck, 2003). The general time reversible model of nucleotide substitution with a gamma-shape parameter and a proportion of invariants (GTR+G+I) was used. For *ppc-2*, two parallel analyses each composed of four chains were run for 20000000 generations, sampling a tree every 1000 generations after a burn-in period of 10000000 generations. Due to slow convergence of parallel runs, the number of parallel chains was increased to sixteen for analyses of *ppc-1*. For this dataset, two different analyses were run for 10000000, sampling a tree each 1000 generations after a burn-in period of 4000000 generations. Consensus trees were computed from all trees sampled after the burn-in period. Convergence of the analyses and adequacy of the burn-in period were determined using Tracer (Rambaut and Drummond, 2007).

In order to represent the rate of amino acid changes, the topologies inferred from the whole *ppc-1* and *ppc-2* datasets were used to infer branch lengths based on amino acid sequences while keeping the topology constant. These analyses were performed in PhyML, under a JTT+G substitution model. Statistical tests of adaptive evolution were also conducted on the group of *ppc-1E1* sequences from Caryophyllales using codon models implemented in codeml of the PAML package (Yang, 2007). These models use the ratio of non-synonymous mutation rate per synonymous mutation rate (ω) as a proxy of selective pressures. An ω smaller than 1 indicates purifying selection, a value of 1 indicates relaxed selection, and a value greater than 1 indicates adaptive evolution. Different models allow ω to vary among sites of the protein or among both sites and branches of the phylogeny (Yang and Nielsen, 2002). The site model without adaptive evolution (M1a) was compared with the site model assuming adaptive evolution on some sites but on all branches (M2a), as well as to several branch-site models assuming adaptive evolution only on some sites and on some branches (referred to as foreground branches; model A). In these branch-site models, foreground branches have to be defined a priori. Different sets of foreground branches were successively selected, and the likelihoods were compared using Akaike information criteria (AIC). In this first model (a), foreground branches were defined as each branch leading to a group of putative C_4_ forms, to the group of sequences belonging to the CAM genera *Mesembryanthemum* and *Drosanthemum*, or to the group of sequences belonging to *Portulaca* and present at high transcript abundance in the night samples (putative CAM form; see Results). In the other models, entire gene lineages present in Portulacineae were successively added to the foreground branches: (b) branches a + *ppc-1E1c*, (c) branches b + *ppc-1E1d* + *ppc-1E1e*, (d) branches c + *ppc-1E1b*, and (e) branches d + *ppc-1E1a*.

Since the evolutionary history of C_4_ photosynthesis in the genus *Portulaca* is debated (Ocampo *et al.*, 2013), the group of *Portulaca* sequences that contains putative C_4_ forms (*ppc-1E1a* and *ppc-1E1a’*) was analysed in more detail. Parallel adaptive genetic changes can bias the phylogenetic reconstruction when they occur in closely related taxa, and considering only third positions of codons can help recover the true phylogenetic relationships (Christin *et al.*, 2007, 2012). To avoid a potential bias due to adaptive evolution, a phylogenetic tree was inferred as described above but considering only third positions of codons from *Portulaca ppc-1E1a* and *ppc-1E1a’* sequences. The inferred topology was used to test alternative scenarios of adaptive evolution using the codon models described above. In addition, to the site models M1a and M2a, different branch-site models (A) were compared. Foreground branches were set following three evolutionary scenarios: adaptive evolution at the base of *Portulaca* (on the branch leading to *ppc-1E1a’*; single C_4_ optimization), at the base of each C_4_ clade of *Portulaca ppc-1E1a’* (multiple C_4_ optimizations); and finally at the base of each C_4_ and C_3_–C_4_ clade of *Portulaca ppc-1E1a’* (multiple C_4_ and C_3_–C_4_ optimizations).

## Results

### PEPC multigene family

The phylogenetic tree inferred using all genes encoding PEPC retrieved from GenBank, the 1KP project, or generated in this study showed that a gene duplication occurred before the emergence of land plants and produced two groups of distantly related genes present in most plant genomes (*ppc-1* and *ppc-2*; Fig. 1; Gowik and Westhoff, 2011). One of these groups (*ppc-1*) contains all the C_4_-specific PEPC genes documented so far (Rao *et al.*, 2002; Svensson *et al.*, 2003; Gowik *et al.*, 2006; Christin *et al.*, 2007, 2011; Besnard *et al.*, 2009; Gowik and Westhoff, 2011). The phylogenetic relationships in each group are compatible with the species relationships predicted from other markers (Supplementary Figs S1 and S2, available at *JXB* online; Soltis *et al.*, 2011). The gene *ppc-2* is present as a single copy in all the species considered (Fig. S2). No gene duplication of *ppc-1* is detectable before the split of eudicots and monocots, but several gene duplications led to the six gene lineages present in grass genomes as shown previously (namely *ppc-aL1a*, *ppc-aL1b*, *ppc-aL2*, *ppc-aR*, *ppc-B1*, and *ppc-B2*; Fig. S1; Christin and Besnard 2009). In addition, a gene duplication occurred soon after the early diversification of eudicots, leading to two gene lineages present in most eudicots and named *ppc-1E1* and *ppc-1E2* (Fig. 1 and Supplementary Fig. S1, available at *JXB* online; these correspond to *ppc-1* and *ppc-2* in Christin *et al.*, 2011).

### Diversification of *ppc-1E1* in Caryophyllales

**Fig. 3.**
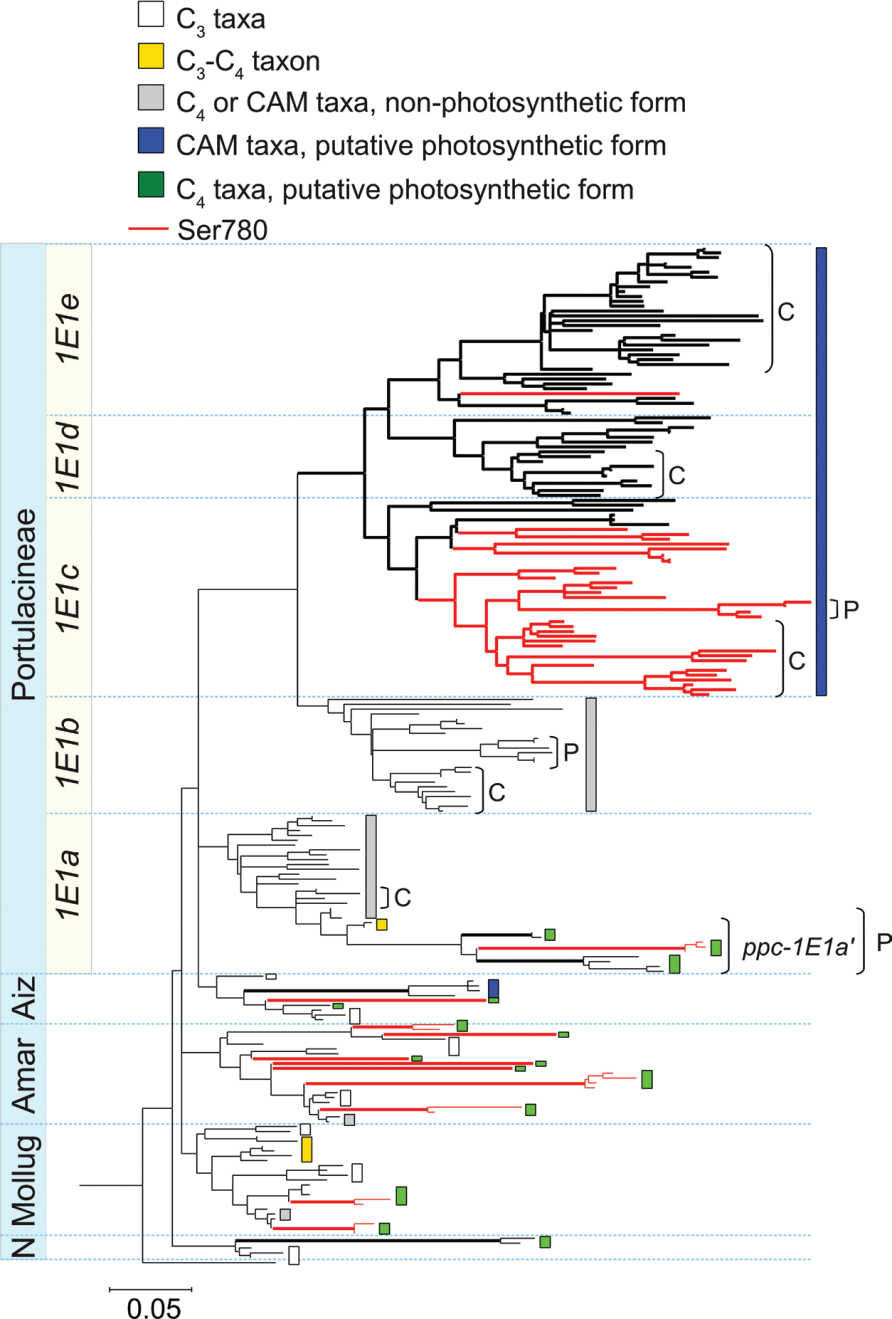
Evolution of *ppc-1E1* in Caryophyllales. The topology was inferred on nucleotide sequences, but branch lengths were estimated based on amino acid sequences. The branch lengths inferred on nucleotide sequences, together with all species names and support values, are available in Fig. S1. Groups of genes encoding a Ser780 are highlighted by red branches. Branches where some sites underwent an excess of non-synonymous mutations according to the best model are thicker. Putative C_4_ forms are delimited in green and putative CAM forms in blue. These were identified based either on transcript abundances in specific conditions, on the literature, or on an excess of amino acid changes in C_4_/CAM species. Genes of C_4_ or CAM taxa that represent putative non-photosynthetic duplicates are delimited in grey, those of C_3_ taxa in white, and those of C_3_–C_4_ taxa in yellow. Families outside Portulacineae and gene lineages for Portulacineae are indicated on the left: N, Nyctaginaceae; Mollug, Molluginaceae; Amar, Amaranthaceae; Aiz, Aizoaceae. Subclades of interest are indicated on the right: P, *Portulaca*; C, cacti. The full phylogenetic tree is available in Fig. S3. The scale bar represents expected substitutions per site. (This figure is available in colour at *JXB* online.)

Genes belonging to the three *ppc* gene lineages present in eudicots were isolated from core Caryophyllales through PCR screening of genomic DNA (Supplementary Tables S1 and S2, available at *JXB* online). The phylogenetic trees of Caryophyllales *ppc-2* and *ppc-1E2* were consistent with published phylogenies based on chloroplast and nuclear markers (Brockington *et al.*, 2009; Arakaki *et al.*, 2011; Kadereit *et al.*, 2012) and no ancient gene duplication was detectable (Supplementary Figs S1 and S2, available at *JXB* online). On the other hand, *ppc-1E1* is present in a high number of copies in members of the Portulacineae (namely *ppc1E1a* to *ppc-1E1e* in Fig. 3 and Supplementary Fig. S1, available at *JXB* online), indicating that this gene lineage was repeatedly duplicated during the early diversification of this clade. The species relationships deduced from each of these gene lineages are consistent with those deduced from chloroplast markers (Arakaki *et al.*, 2011). The *ppc-1E1a* gene lineage includes sequences isolated previously from cDNA of two members of Portulacineae, *Pereskia aculatea* and *Selenicereus vitii* (Gehrig *et al.*, 2001).

The *ppc-1E1* lineage contains genes encoding putative C_4_-specific forms in *Alternanthera* (Gowik *et al.*, 2006), *Bienertia* and *Suaeda* (Lara *et al.*, 2006), as well as genes encoding putative CAM-specific forms in *Mesembryanthemum* (Rickers *et al.*, 1989). Most C_4_-specific PEPC genes studied previously encode a serine residue at the position homologous to position 780 of the maize PEPC protein (numbering based on *Zea mays* sequence CAA33317; hereafter referred to as Ser780), although this Ser780 is not necessary for the C_4_ function (Rao *et al.*, 2008). In *Flaveria*, Ser780 has been shown to be a major determinant of the C_4_ properties of PEPC (Bläsing *et al.*, 2000; Engelmann *et al.*, 2002; Svensson *et al.*, 2003). The homologous position is occupied by a conserved alanine in all characterized PEPC genes not involved in C_4_ photosynthesis (Svensson *et al.*, 2003; Christin *et al.*, 2007, 2011; Besnard *et al.*, 2009). In the present dataset, a Ser780 is encoded by *ppc-1E1* genes from the C_4_ taxa in Amaranthaceae, Aizoaceae, and Molluginaceae, while it is not encoded by homologous genes of C_3_ taxa of the same families and from other gene lineages (Fig. 3 and Supplementary Figs S1 and S2, available at *JXB* online). This supports earlier suggestions that *ppc-1E1* encodes the C_4_-specific PEPC in numerous C_4_ Caryophyllales (Gowik *et al.*, 2006; Christin *et al.*, 2011; Gowik and Westhoff, 2011). However, the Ser780 is not encoded by *ppc-1E1* genes from some C_4_ taxa in Nyctaginaceae and Aizoaceae.

Most members of Portulacineae have the ability to perform some degree of CAM (Guralnick *et al.*, 2008; Nyffeler *et al.*, 2008). The *ppc-1E1c* genes of Portulacineae encode a Ser780 in most species (Fig. 3). Sequences isolated by PCR from cDNA extracted from the stems of *Nopalea* and *Echinocereus* at night belong to this gene lineage and encode a Ser780, which indicates that proteins encoded by *ppc-1E1c* might be involved in the CAM pathway of cacti. This is further supported by the isolation of *ppc-1E1c* sequences from cDNA of another cactus (*Hylocereus undatus*; NCBI accession JF966382). However, the Ser780 residue is not encoded by *ppc-1E1c* genes in the sampled Montiaceae and in several of the sampled Didiereaceae (Supplementary Fig. S1, available at *JXB* online). In addition, the *ppc-1E1e* from *Ceraria* (Didiereaceae; Supplementary Fig. S1, available at *JXB* online) also encodes a Ser780. All other Portulacineae *ppc-1E1* gene copies and all *ppc-2* and *ppc-1E2* (with the exception of some sequences from *Flaveria* known to be involved in C_4_ photosynthesis; Svensson *et al.*, 2003) encode the Ala780 typical of non-C_4_ genes.

### Transcriptome from *Portulaca oleracea*

Differences in estimates of transcript abundance of C_4_-related genes between individuals grown under the same watering regime were small (Supplementary Table S6, available at *JXB* online). In the well-watered samples, some of the gene lineages encoding the enzymes of the C_4_ pathway (β-CA, PEPC, NAD-MDH, NADP-MDH, ALA-AT, ASP-AT, PPDK, NAD-ME and NADP-ME) were present at high transcript abundance during the day (Supplementary Table S6, available at *JXB* online). With the exception of NADP-ME, these correspond to the enzymes postulated to play a role in the C_4_ pathway of *P*. *oleracea* (Lara *et al.*, 2004). After 2h in the dark, the transcript abundance of these enzymes remained substantial, although in most cases it was strongly reduced compared with the day sample (Supplementary Table S6, available at *JXB* online). The only exception is the gene for ALA-AT, which was present at higher transcript abundance in the dark than in the light. In addition, one of the individuals showed a slight increase in the transcript abundance of one of the genes for NADP-MDH at night while its abundance was extremely low in the other individual (Supplementary Table S6, available at *JXB* online).

In the samples watered less frequently, genes encoding the same enzymes were present at high transcript abundance during the day, although the levels were generally lower than in the well-watered sample (Supplementary Table S6, available at *JXB* online). At night, genes encoding all C_4_-related enzymes were present at lower abundance, with the exception of genes for PEPC and NADP-MDH, for which the abundance of one gene lineage increased to reach levels equivalent to, or even higher than, those observed for the well-watered samples during the day (Table 1 and Supplementary Table S6, available at *JXB* online). This is consistent with the nocturnal part of the purported CAM cycle of *P*. *oleracea*, which is based on these two enzymes (Lara *et al.*, 2004). However, the nocturnal part was assumed to also involve carbonic anhydrase, but the transcript abundance of this enzyme is strongly reduced at night although it remains high (Supplementary Table S6, available at *JXB* online). The transcript abundances suggest that a C_4_ cycle is present in both well-watered and drought conditions, but it is complemented by a CAM cycle in drought conditions, as indicated previously (Mazen, 2000).

**Table 1.** Transcript abundances in rpm of PEPC-encoding genes in Portulaca oleracea grown in different conditions

Time	Day		Night		Day		Night	
Condition	Watered frequently				Watered occasionally			
Individual	1	2	1	2	3	4	3	4
*ppc-2*	13	14	6	1	5	30	7	17
*ppc-1E2*	44	41	40	29	14	10	5	14
*ppc-1E1a*	131	139	157	192	167	196	185	173
*ppc-1E1b*	0	1	0	7	6	18	0	0
*ppc-1E1c*	3	0	820	1180	277	338	7602	6823
*pp c-1E1a’*	9916	4697	6710	6052	7339	1868	1421	869

### Detailed analysis of *P. oleracea* PEPC genes

The incorporation of contigs from the *P*. *oleracea* samples into the densely sampled Caryophyllales dataset allowed us to estimate the transcript abundance of each *ppc-1E1* gene lineage (Table 1). In addition to *ppc-1E2* and *ppc-2*, four distinct *ppc-1E1* genes were isolated from the transcriptomes of *P*. *oleracea*, only one of which was also isolated from genomic DNA (*ppc-1E1b*). One of these four genes was clearly nested within *ppc-1E1c* and two in *ppc-1E1a* (Supplementary Fig. S1, available at *JXB* online). The phylogenetic relationships suggest a recent duplication of *ppc-1E1a* in *Portulaca* and one of the duplicates was named *ppc-1E1a’*.

The genes *ppc-1*, *ppc-2*, *ppc-1E1a*, and *ppc-1E1b* were present at similarly low abundances in all samples (Table 1). By contrast, *ppc-1E1a’* was present at very high transcript abundances during the day in the well-watered samples (Table 1). This pattern is consistent with a function in the C_4_ pathway, which is moreover supported by the Ser780 encoded by the gene. The abundance of *ppc-1E1a’* during the day decreased in one of the individuals that were watered less frequently and its abundance at night decreased in both individuals (Table 1). The gene *ppc-1E1c* was present at extremely low transcript abundances in the well-watered samples during the day. However, its abundance increased at night, and the nocturnal abundance was considerably higher in infrequently watered than in well-watered plants (Table 1). High nocturnal transcript abundance triggered by reduced water availability supports an involvement of the encoded enzyme in the CAM pathway of *P*. *oleracea*. This gene also encodes a Ser780.

### Distribution of *ppc-1E1* genes in other *Portulaca* species and evidence of adaptive evolution

**Fig. 4.**
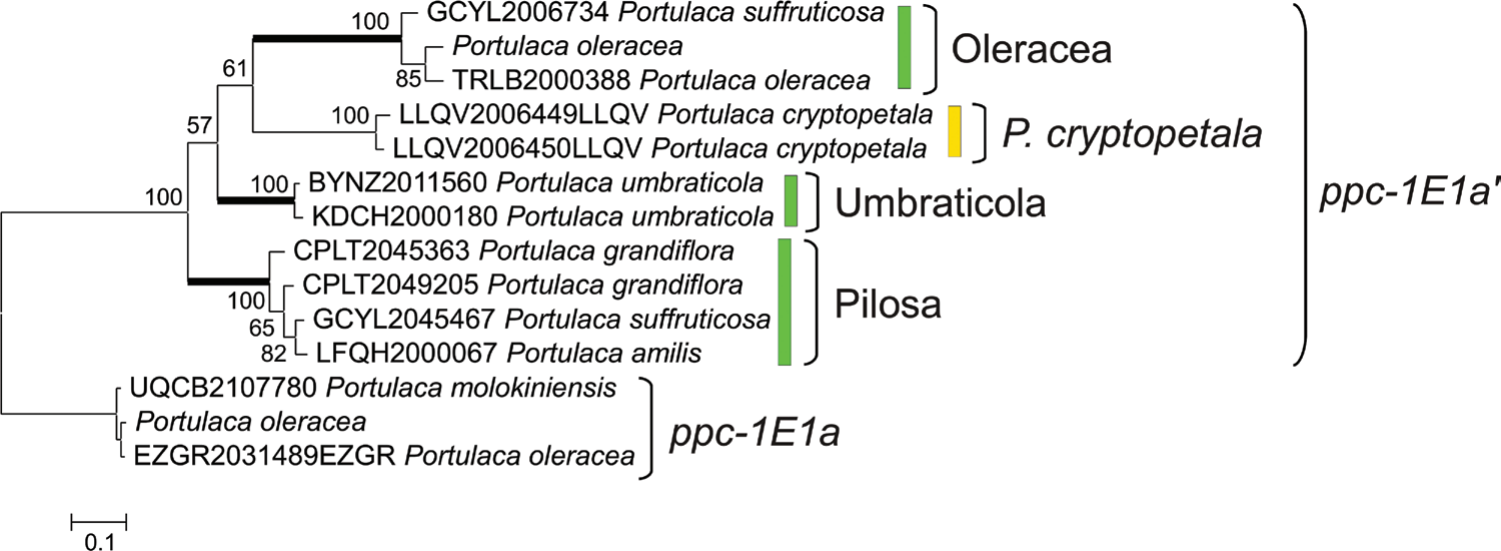
Evolution of C_4_-specific PEPC genes in *Portulaca*. This phylogeny of *Portulaca ppc-1E1a*/*ppc-1E1a’* was inferred from third positions of codons. Bayesian support values are indicated near branches and clades are delimited on the right. Putative C_4_ forms are highlighted in green and putative C_3_–C_4_ forms in yellow. Thick branches represent inferred episodes of adaptive evolution. The scale bar represents expected substitutions per site. (This figure is available in colour at *JXB* online.)

Using the 1KP transcriptome data, sequences corresponding to the genes *ppc-1E1a* and *ppc-1E1c* were retrieved from the Oleracea clade, while sequences corresponding to *ppc-1E1b* were retrieved from both Oleracea and Pilosa clades (Supplementary Fig. S1, available at *JXB* online). The RNA for this project was isolated during the day and putative CAM-specific genes would likely be missed. The putative C_4_ gene *ppc-1E1a’* was retrieved from the four *Portulaca* clades. One sequence attributed to *P*. *suffruticosa* was nested within *ppc-1E1a’* of the Pilosa clade (Fig. 4 and Supplementary Fig. S1, available at *JXB* online), which might indicate a biologically relevant phenomenon (e.g. hybridization) or a methodological problem (e.g. cross-contamination). Since these sequences were retrieved from leaf RNA isolated during the day, the presence of transcripts corresponding to *ppc-1E1a’* is compatible with the hypothesis that this gene is involved in C_4_ photosynthesis of these different species.

The branch lengths estimated from amino acid sequences strongly vary among clades (Fig. 3 and Supplementary Fig. S3, available at *JXB* online). Since the variation is more pronounced than with nucleotide sequences (Supplementary Figs S1 and S2, available at *JXB* online), it indicates an excess of non-synonymous mutations. In Molluginaceae and Amaranthaceae, clear increases in the rate of amino acid substitutions occurred on branches leading to genes encoding putative C_4_-specific enzymes. In Aizoaceae, a similar acceleration is visible on the branch leading to the putative C_4_ gene of *Trianthema*, but also to the genes of the CAM plant *Mesembryanthemum crystallinum*. In addition, the branch leading to the C_4_ Nyctaginaceae *Boerhavia* underwent many amino acid changes (Fig. 3), which is suggestive of functional divergence, potentially as an adaptation to the C_4_ context. These *ppc-1E1* genes likely encode proteins involved in CCMs, but do not encode the Ser780. This indicates that changes happened in different parts of the coding sequences. The monophyletic group composed of Portulacineae *ppc-1E1c*, *ppc-1E1d*, and *ppc-1E1e* is also characterized by increased rates of amino acid substitutions, with further increases in some branches, such as those leading to *ppc-1E1c* from *Portulaca*, but also the cacti (Fig. 3 and Supplementary Fig. S3, available at *JXB* online). Within Portulacineae *ppc-1E1a*’, long branches lead to the putative C_4_-specific genes of *Portulaca*. However, most of the amino acid substitutions occurred after the divergence of the four clades (*sensu* Ocampo and Columbus, 2012) and comparatively few happened on the branch leading to the C_3_–C_4_ *P*. *cryptopetala* (Fig. 3).

The action of adaptive evolution on some branches of the phylogeny is supported by codon models. While the model assuming positive selection on some sites but all branches of Caryophyllales *ppc-1E1* was not better than the null model, assuming increased rates of non-synonymous changes on some sites but some branches only led to a very significant increase in likelihood (Table 2). While all the tested sets of foreground branches led to a significant increase of likelihood, the model assuming increased rates of non-synonymous mutations in the whole of Portulacineae *ppc-1E1c*, *ppc-1E1d*, and *ppc-1E1e* gene lineages in addition to C_4_ and CAM clades outside of Portulacineae produced the best AIC (Table 2). In this model, 16.8% of sites were estimated to undergo more non-synonymous mutations in the selected foreground branches, although the optimized *dN*/*dS* ratio was not different from 1.

**Table 2.** Codon models for ppc-1E1 of Caryophyllales

Model	Foreground branches	Number of parameters	Log- likelihood	AIC score
M1a^*a*^	–	399	–49702	10202
M2a^*b*^	–	401	–49702	10206
A^*c*^	(a) each C_4_ and CAM groups^*d*^	401	–49588	99978
A^*c*^	(b) a + *ppc-1E1c*	401	–49523	99848
A^*c*^	(c) b + *ppc-1E1d* + *ppc-1E1e*	401	–49383	99568
A^*c*^	(d) c + *ppc-1E1b*	401	–49416	99634
A^*c*^	(e) d + *ppc-1E1a*	401	–49556	99914

^*a*^ Site model without adaptive evolution.

^*b*^ Site model with adaptive evolution.

^*c*^ Branch-site model with adaptive evolution.

^*d*^ Except for Portulacineae other than *Portulaca*.

In the phylogenetic tree inferred from all nucleotides, *P*. *cryptopetala ppc-1E1a’* is sister to all other *Portulaca* (Supplementary Fig. S1, available at *JXB* online), but this species is sister to the Oleracea clade in the tree inferred on third positions of codons (Fig. 4), as expected based on other markers (Ocampo and Columbus, 2012; Ocampo *et al.*, 2013). The model assuming adaptive evolution in the entire *Portulaca ppc-1E1a*/*ppc-1E1a’* clade was not different from the model without adaptive selection. Similarly, assuming adaptive evolution at the base of *ppc-1E1a’* did not improve the likelihood. However, assuming adaptive evolution at the base of each C_4_ clade of *ppc-1E1a’* significantly improved the model (χ^2^=38.2, df=2, *P* <0.00001), which indicates that 10.7% of the sites have evolved under adaptive evolution on these branches, with a *dN*/*dS* ratio of 1.35. The model assuming adaptive evolution on branches leading to both C_4_ and C_3_–C_4_ clades was also better than the null model, but not as good as the model without adaptive evolution on the C_3_–C_4_ branch (difference of AIC >18).

## Discussion

### Increased rates of amino acid changes in both C_4_ and CAM origins

The evolution of genes encoding PEPC in the Caryophyllales is characterized by increased rates of amino acid substitutions and the recurrence of several amino acid changes previously detected in C_4_ monocots (e.g. E572Q, H665N, and A780S; Christin *et al.*, 2007; Besnard *et al.*, 2009). These increased rates of amino acid change are not limited to C_4_ taxa, but are also observed in CAM lineages, including Aizoaceae and Portulacineae species (Fig. 3), and the excess of non-synonymous mutations on these branches was confirmed with codon models (Table 2). C_4_- and CAM-specific PEPC differ in the timing of their activity, but the catalytic challenges they face in the two cycles are similar, because in both cases the concentrations of both substrates and products are greatly increased. The evolution of both C_4_- and CAM-specific PEPC consequently required adaptive mutations, some of which are shared among multiple origins, while several are probably specific to one or a few clades and might depend on the other amino acid mutations undergone by the coding sequence of the gene before its co-option.

In Portulacineae, the *ppc-1E1* gene lineage is present in five copies, which appeared through several rounds of gene duplications (Fig. 3 and Supplementary Fig. S1, available at *JXB* online). The three most recent copies, namely *ppc-1E1c*, *ppc-1E1d*, and *ppc-1E1e*, are all characterized by increased rates of amino acid substitutions (Fig. 3 and Table 2). The Portulacineae encompass species with different degrees of CAM metabolism (Guralnick and Jackson, 2001; Nyffeler *et al.*, 2008). The high number of *ppc-1E1* copies could have promoted neofunctionalization of the genes by relaxing selective constraints, facilitating the diversification of photosynthetic types in this group. A gradual upregulation of the CCM over time would have triggered successive periods of adaptive genetic changes in response to modifications of the catalytic environment, explaining the high rates of amino acid substitutions sustained in the entire clade (Fig. 3). For instance, the accumulation of mutations on the branches leading to *ppc-1E1c* of CAM-constitutive cacti (Cactoideae and Opuntioideae; Fig. 3) could be linked to the evolution of a more efficient CAM pathway in these taxa. This contrasts with the evolution of C_4_-specific PEPC where adaptive changes are concentrated at the base of each C_4_ group (Fig. 3; Christin *et al.*, 2007; Besnard *et al.*, 2009), and might indicate that the optimization of PEPC for the CAM function is spread over a longer time period.

### C_4_ origins in *Portulaca* within a CAM-like context

The putative gene encoding the CAM-specific PEPC in *Portulaca* belongs to the *ppc-1E1c* gene lineage (Fig. 3). This gene lineage is characterized by increased rates of amino acid substitutions in other CAM taxa, such as cacti. This suggests that members of this *ppc-1E1c* gene lineage may have been already involved in some type of CAM metabolism before the divergence of *Portulaca* and cacti. On the other hand, the putative C_4_-specific genes of *Portulaca* belong to the *ppc-1E1a* gene lineage, which is duplicated in these taxa. One of the duplicates was likely co-opted for C_4_ photosynthesis after the gene duplication. Other members of the *ppc-1E1a* gene lineage, including the second duplicate of *Portulaca*, underwent mutations that generated amino acid substitutions at the same rate as genes from C_3_ species (Fig. 3). Codon models confirmed that adaptive non-synonymous mutations did not occur on these genes, but were restricted to some members of the *ppc-1E1a*′ duplicate, which is specific to *Portulaca*. The evolution of C_4_-specific genes in *Portulaca* likely co-opted a non-CCM gene through numerous changes in the coding sequences. Therefore, the distribution of high rates of non-synonymous substitutions indicates that the evolution of C_4_-specific PEPC occurred after the divergence of *Portulaca* from other Portulacineae while the CAM-specific properties of the gene used by *Portulaca* were inherited from the common ancestor of *Portulaca* and the cacti.

Because *Portulaca* species are nested within a predominantly CAM lineage (Guralnick and Jackson, 2001; Nyfeller *et al.*, 2008), it has been previously hypothesized that the C_4_ pathway of these taxa evolved from an ancestral CAM type (Sage, 2002). This hypothesis is corroborated by the evolutionary history of PEPC genes, with the CAM-specific gene of *Portulaca* being similar to CAM forms of other species, such as cacti (Fig. 3), while the C_4_-specific PEPC has been recruited from non-photosynthetic forms. For the other enzymes of the CAM and C_4_ pathways, *Portulaca* uses the same genes in both conditions (Supplementary Table S6, available at *JXB* online). This indicates that, for many enzymes of the CCM, the evolution of one CCM from the other does not require the co-option of new genes. However, since the timing of activity differs between the CCMs, modifications in the regulation of the genes are probably still required. For many of these genes, this may be possible because they are involved with both the C_3_ pathway and the decarboxylation phase of the C_4_ pathway that operates during the day in both the CAM and C_4_ pathways. In the case of PEPC, however, co-option of the CAM-specific gene into the C_4_ cycle might have been hampered by the distinct regulatory cascades controlling the transcription and translation of the CAM form at night and the C_4_ form during the day (Jiao and Chollet, 1991; Nimmo, 2003), leading to the recruitment of a distinct gene (namely *ppc-1E1a’*).

### C_4_ evolution within *Portulaca*

*Portulaca* species do not form a homogeneous C_4_ group, but include a C_3_–C_4_ species and several C_4_ clades that differ in their C_4_-associated anatomical types and decarboxylating enzymes used for the C_4_ cycle (Voznesenskaya *et al.*, 2010; Ocampo *et al.*, 2013). In phylogenetic trees of *Portulaca* species, the C_3_–C_4_ taxon is nested within otherwise C_4_ lineages (Ocampo and Columbus, 2012), which might be interpreted as evidence for a C_4_ to C_3_–C_4_ reversion (Ocampo *et al.*, 2013). However, evolutionary transitions between photosynthetic types are difficult to reconstruct based on species relationships, and C_4_-related phenotypic and genetic variation can help differentiate alternative scenarios (Christin *et al.*, 2010; Hancock and Edwards, 2014). In the case of *Portulaca*, the PEPC gene of the C_3_–C_4_ *P*. *cryptopetala* is nested within those of different C_4_ clades (Fig. 4). If PEPC had been optimized once for a C_4_ function at the base of *Portulaca*, this would have occurred through adaptive evolution on the branch sustaining the whole clade. Such a scenario is ruled out, however, by modelling of codon transitions, which strongly favour a model with adaptive evolution restricted to the branches at the base of each of the three C_4_ clades (Fig. 4). This shows that the putative C_4_-specific PEPC of the three C_4_ clades included in this study underwent adaptive amino acid changes after their divergence, and after their separation from the lineage of *P*. *cryptopetala*. For instance, the Ser780 is restricted to genes from the Oleracea clade, while orthologous genes from members of the Pilosa clade underwent other amino acid substitutions that are shared with C_4_ monocots (e.g. A531P, S761A; Christin *et al.*, 2007). These results show that the optimization of PEPC for a function in C_4_ photosynthesis occurred independently in each C_4_ clade, and refutes a C_4_ to C_3_–C_4_ reversal in *P*. *cryptopetala*.

In addition to independent optimizations of PEPC genes for C_4_ photosynthesis, variation exists in the C_4_-associated anatomy and biochemistry among C_4_ clades of *Portulaca* (Voznesenskaya *et al.*, 2010; Ocampo *et al.*, 2013). Based on these observations and our results, the most likely scenario is the addition of a C_3_–C_4_ suite of traits over an ancestral CAM-like type in the common ancestor of *Portulaca*. This C_3_–C_4_ type might have been co-opted several times independently for the evolution of a more efficient C_4_ trait, as suggested for Molluginaceae (Christin *et al.*, 2011). A gradual increase of PEPC activity during the day might then have occurred concomitantly with the development of a more C_4_-like anatomy, characterized by a high bundle sheath to mesophyll ratio. One way to achieve this state is through high vein densities. Some members of *Portulaca* belong to a handful of lineages in the Portulacineae to have evolved high vein densities via the rearrangement of leaf vasculature into a three-dimensional configuration (Voznesenskaya *et al.*, 2010; Ocampo *et al.*, 2013; Ogburn and Edwards, 2013). While most of these vein rearrangements were associated with large increases in succulence, in *Portulaca* it may have allowed the acquisition of an optimized C_4_ CCM.

## Conclusions

Caryophyllales is a hotspot of photosynthetic transitions, with at least 23 C_4_ and multiple CAM origins. Of three PEPC gene lineages present in eudicots (*ppc-1E1*, *ppc-1E2*, and *ppc-2*; Fig. 1), only *ppc-1E1* was recurrently recruited into the C_4_ pathway, suggesting that this gene lineage was more suitable for a C_4_ function (Christin *et al.*, 2013a). The evidence provided here also supports recruitment of the same gene lineage into CAM metabolism, which suggests that the same capacitated genes present in the common C_3_ ancestor were co-opted by the numerous CCM origins of Caryophyllales. The evolvability of one CCM compared to the other might depend on the ecology and leaf anatomy of the C_3_ ancestor of each lineage (Sage, 2002; Edwards and Ogburn, 2012; Edwards and Donoghue, 2013). However, in some cases, evolutionary bridges between the two photosynthetic types exist, as illustrated by *Portulaca*. This shows that while ancestral conditions could influence the evolutionary trajectories of the descendants, the determinism is not perfect and one photosynthetic type can be co-opted to evolve the other.

## Supplementary material

Supplementary data are available at *JXB* online.

Figure S1. Phylogenetic relationships among *ppc-1* genes. This phylogenetic tree was obtained through Bayesian inference on nucleotide sequences. Names of taxonomic groups and gene lineages are indicated on the right. Branches in lineages presenting a Ser780 are highlighted in red. Bayesian support values are indicated near branches. Asterisks indicate putative pseudogenes with one or several stop codons in the coding sequence. Black circles indicate sequences that were isolated from cacti cDNA. (A) Complete phylogenetic tree; (B) *ppc-1E2* of Caryophyllales; (C, D) *ppc-1E1* of Caryophyllales.

Figure S2. Phylogenetic relationships among *ppc-2* genes. This phylogenetic tree was obtained through Bayesian inference on nucleotide sequences. Names of taxonomic groups are indicated on the right. Bayesian support values are indicated near branches.

Figure S3. Amino acid changes on genes encoding PEPC. The topology was inferred on nucleotide sequences, but branch lengths were estimated based on amino acid sequences. The branch lengths inferred on nucleotide sequences, together with all species names and support values, are available in Figs S1 and S2. The names of the main groups are indicated on the right. Groups of genes containing a Ser780 are highlighted by red branches. The asterisk highlights a pseudogene with multiple stop codons.

Table S1. Sample of Caryophyllales (excluding Portulacineae) used for analyses of PEPC-encoding genes.

Table S2. List of Portulacineae genes encoding PEPC analysed.

Table S3. Additional primers used for PCR amplification of Caryophyllales genes encoding PEPC.

Table S4. Water treatment of *Portulaca oleracea* plants.

Table S5. Sequencing and mapping statistics.

Table S6. Expression levels in rpm of C_4_-related genes in day and night samples of *Portulaca oleracea* plants grown in different conditions.

Supplementary Data

## Abbreviations:

AIC: Akaike information criteria
CCM: CO_2_-concentrating mechanism
PEPC: phosphoenolpyruvate carboxylase
rpm: reads per million of alignable reads.

## References

[CIT0001] Arakaki M Christin PA Nyffeler R Lendel A Eggli U Ogburn RM Spriggs E Moore MJ Edwards EJ 2011 Contemporaneous and recent radiations of the world’s major succulent plant lineages. Proceedings of the National Academy of Sciences USA 108, 8379–8384 10.1073/pnas.1100628108 PMC3100969 21536881

[CIT0002] Aubry S Brown NJ Hibberd JM 2011 The role of proteins in C_3_ plants prior to their recruitment into the C_4_ pathway. Journal of Experimental Botany 62, 3049–3059 21321052 10.1093/jxb/err012

[CIT0003] Bauwe H Chollet R 1986 Kinetic properties of phosphoenolpyruvate carboxylase from C_3_, C_4_, and C_3_–C_4_ intermediate species of *Flaveria* (Asteraceae). Plant Physiology 82, 695–699 16665094 10.1104/pp.82.3.695 PMC1056191

[CIT0004] Besnard G Muasya AM Russier F Roalson EH Salamin N Christin PA 2009 Phylogenomics of C_4_ photosynthesis in sedges (Cyperaceae): multiple appearances and genetic convergence. Molecular Biology and Evolution 26, 1909–1919 19461115 10.1093/molbev/msp103

[CIT0005] Bläsing OE Westhoff P Svensson P 2000 Evolution of C_4_ phosphoenolpyruvate carboxylase in *Flaveria*, a conserved serine residue in the carboxyl-terminal part of the enzyme is a major determinant of C_4_-specific characteristics. Journal of Biological Chemistry 275, 27917–27923 10871630 10.1074/jbc.M909832199

[CIT0006] Borland AM Taybi T 2004 Synchronization of metabolic processes in plants with Crassulacean acid metabolism. Journal of Experimental Botany 55, 1255–1265 15073222 10.1093/jxb/erh105

[CIT0007] Bräutigam A Kajala K Wullenweber J 2011 An mRNA blueprint for C_4_ photosynthesis derived from comparative transcriptomics of closely related C_3_ and C_4_ species. Plant Physiology 155, 142–156 20543093 10.1104/pp.110.159442 PMC3075794

[CIT0008] Brockington SF Alexandre R Ramdial J Moore MJ Crawley S Dhingra A Kilu K Soltis DE Soltis PS 2009 Phylogeny of the Caryophyllales sensu lato: revisiting hypotheses on pollination biology and perianth differentiation in the core Caryophyllales. International Journal of Plant Sciences 170, 627–643

[CIT0009] Christin PA Besnard G 2009 Two independent C_4_ origins in Aristidoideae (Poaceae) revealed by the recruitment of distinct phosphoenolpyruvate carboxylase genes. American Journal of Botany 96, 2234–2239 21622339 10.3732/ajb.0900111

[CIT0010] Christin PA Besnard G Edwards EJ Salamin N 2012 Effect of genetic convergence on phylogenetic inference. Molecular Phylogenetics and Evolution 62, 921–927 22197805 10.1016/j.ympev.2011.12.002

[CIT0011] Christin PA Boxall SF Gregory R Edwards EJ Hartwell J Osborne CP 2013a Parallel recruitment of multiple genes into C_4_ photosynthesis. Genome Biology and Evolution 5, 2174–2187 24179135 10.1093/gbe/evt168 PMC3845648

[CIT0012] Christin PA Freckleton RP Osborne CP 2010 Can phylogenetics identify C_4_ origins and reversals? Trends in Ecology and Evolution 25, 403–409 20605250 10.1016/j.tree.2010.04.007

[CIT0013] Christin PA Osborne CP 2013 The recurrent assembly of C_4_ photosynthesis, an evolutionary tale. Photosynthesis Research 117, 163–175 23703454 10.1007/s11120-013-9852-z

[CIT0014] Christin PA Osborne CP Chatelet DS Columbus JT Besnard G Hodkinson TR Garrison LM Vorontsova MS Edwards EJ 2013b Anatomical enablers and the evolution of C_4_ photosynthesis in grasses. Proceedings of the National Academy of Sciences USA 110, 1381–1386 10.1073/pnas.1216777110 PMC3557070 23267116

[CIT0015] Christin PA Sage TL Edwards EJ Ogburn RM Khoshravesh R Sage RF 2011 Complex evolutionary transitions and the significance of C_3_–C_4_ intermediate forms of photosynthesis in Molluginaceae. Evolution 65, 643–660 20955197 10.1111/j.1558-5646.2010.01168.x

[CIT0016] Christin PA Salamin N Savolainen V Duvall MR Besnard G 2007 C_4_ photosynthesis evolved in grasses via parallel adaptive genetic changes. Current Biology 17, 1241–1247 17614282 10.1016/j.cub.2007.06.036

[CIT0017] Crayn DM Winter K Smith JAC 2004 Multiple origins of crassulacean acid metabolism and the epiphytic habit in the Neotropical family Bromeliaceae. Proceedings of the National Academy of Sciences USA 101, 3703–3708 10.1073/pnas.0400366101 PMC373526 14982989

[CIT0018] Dong LY Masuda T Kawamura T Hata S Izui K 1998 Cloning, expression, and characterization of a root-form phosphoenolpyurvate carboxylase from *Zea mays*: comparison with the C_4_-form enzyme. Plant and Cell Physiology 39, 865–873 9787461 10.1093/oxfordjournals.pcp.a029446

[CIT0019] Dunn CW Howison M Zapata F 2013 Agalma: an automated phylogenomics workflow. BMC Bioinformatics 14, 300 24252138 10.1186/1471-2105-14-330 PMC3840672

[CIT0020] Edgar RC 2004 MUSCLE: multiple sequence alignment with high accuracy and high throughput. Nucleic Acids Research 32, 1792–1797 15034147 10.1093/nar/gkh340 PMC390337

[CIT0021] Edwards EJ Donoghue MJ 2013 Is it easy to move *and* easy to evolve? Evolutionary accessibility and adaptation. Journal of Experimental Botany 64, 4047–4052 23913955 10.1093/jxb/ert220

[CIT0022] Edwards EJ Ogburn RM 2012 Angiosperm responses to a low-CO_2_ world: CAM and C_4_ photosynthesis as parallel evolutionary trajectories. International Journal of Plant Sciences 173, 724–733

[CIT0023] Engelmann S Bläsing OE Westhoff P Svensson P 2002 Serine 774 and amino acids 296 to 437 comprise the major C_4_ determinants of the C_4_ phosphoenolpyruvate carboxylase of *Flaveria trinervia* . FEBS Letters 524, 11–14 12135733 10.1016/s0014-5793(02)02975-7

[CIT0024] Finnegan PM Suzuki S Ludwig M Burnell JN 1999 Phosphoenolpyruvate carboxykinase in the C_4_ monocot *Urochloa panicoides* is encoded by four differentially expressed genes. Plant Physiology 120, 1033–1041 10444086 10.1104/pp.120.4.1033 PMC59336

[CIT0025] Gehrig H Heute V Kluge M 2001 New partial sequences of phosphoenolpyruvate carboxylase as molecular phylogenetic markers. Molecular Phylogenetics and Evolution 20, 262–274 11476634 10.1006/mpev.2001.0973

[CIT0026] Goodstein DM Shu S Howson R 2012 Phytozome: a comparative platform for green plant genomics. Nucleic Acids Research 40, D1178–D1186 22110026 10.1093/nar/gkr944 PMC3245001

[CIT0027] Gowik U Bräutigam A Weber KL Weber APM Westhof P 2011 Evolution of C_4_ photosynthesis in the genus *Flaveria*: how many and which genes does it take to make C_4_? Plant Cell 23, 2087–2105 21705644 10.1105/tpc.111.086264 PMC3160039

[CIT0028] Gowik U Engelmann S Bläsing OE Raghavendra AS Westhoff P 2006 Evolution of C_4_ phosphoenolpyruvate carboxylase in the genus *Alternanthera*: gene families and the enzymatic characteristics of the C_4_ isozyme and its orthologues in C_3_ and C_3_/C_4_ Alternantheras. Planta 223, 359–368 16136331 10.1007/s00425-005-0085-z

[CIT0029] Gowik U Westhoff P 2011 C_4_-phosphoenolpyruvate carboxylase. In: Rhagavendra AS Sage RF, eds. C_4_ photosynthesis and related CO_2_ concentrating mechanisms. Advances in Photosynthesis and Respiration, *Vol* 32 Dordrecht: Springer, 257–275

[CIT0030] Grabherr MG Haas BJ Yassour M 2011 Full-length transcriptome assembly from RNA-seq data without a reference genome. Nature Biotechnology 29, 644–652 10.1038/nbt.1883 PMC3571712 21572440

[CIT0031] Griffiths H Weller G Toy LMF Dennis RJ 2013 You’re so vein: bundle sheath physiology, phylogeny and evolution in C_3_ and C_4_ plants. Plant Cell and Environment 36, 249–261 10.1111/j.1365-3040.2012.02585.x 22827921

[CIT0032] Guindon S Gascuel O 2003 A simple, fast, and accurate algorithm to estimate large phylogenies by maximum likelihood. Systematic Biology 52, 696–704 14530136 10.1080/10635150390235520

[CIT0033] Guralnick LJ Cline A Smith M Sage RF 2008 Evolutionary physiology: the extent of C_4_ and CAM photosynthesis in the genera *Anacampseros* and *Grahamia* of the Portulacaceae. Journal of Experimental Botany 59, 1735–1742 18440927 10.1093/jxb/ern081

[CIT0034] Guralnick LJ Edwards G Ku MSB Hockema B Franceschi VR 2002 Photosynthesis and anatomical characteristics in the C_4_-crassulacean acid metabolism-cycling plant *Portulaca grandiflora* . Functional Plant Biology 29, 763–773 10.1071/PP01176 32689524

[CIT0035] Guralnick LJ Jackson M 2001 The occurrence and phylogenetics of CAM in the Portulacaceae. International Journal of Plant Sciences 162, 257–262

[CIT0036] Guralnick LJ Ting IP 1987 Physiological changes in *Portulacaria afra* (L.) Jacq. during a summer drought and rewatering. Plant Physiology 85, 481–486 16665724 10.1104/pp.85.2.481 PMC1054282

[CIT0500] Hancock L Edwards EJ 2014 Phylogeny and the inference of evolutionary trajectories. Journal of Experimental Botany 65, 3491–3498 10.1093/jxb/eru118 PMC4085962 24755279

[CIT0037] Hatch MD 1987 C_4_ photosynthesis: a unique blend of modified biochemistry, anatomy and ultrastructure. Biochimica et Biophysica Acta 895, 81–106

[CIT0038] Hattersley PW 1984 Characterization of C_4_ type leaf anatomy in grasses (Poaceae), mesophyll-bundle sheath area ratios. Annals of Botany 53, 163–179

[CIT0039] Jiao JA Chollet R 1991 Posttranslational regulation of phosphoenolpyruvate carboxylase in C_4_ and Crassulacean Acid Metabolism plants. Plant Physiology 95, 981–985 16668131 10.1104/pp.95.4.981 PMC1077640

[CIT0040] Johnson MTJ Carpenter EJ Tian Z 2012 Evaluating methods for isolating total RNA and predicting the success of sequencing phylogenetically diverse plant transcriptomes. PLOS ONE 7, e50226 23185583 10.1371/journal.pone.0050226 PMC3504007

[CIT0041] Kadereit G Ackerly D Pirie MD 2012 A broader model for C_4_ photosynthesis evolution in plants inferred from the goosefoot family (Chenopodiaceae s.s.). Proceedings of the Royal Society of London Series B 279, 3304–3311 22628474 10.1098/rspb.2012.0440 PMC3385724

[CIT0042] Kadereit G Borsch T Weising K Freitag H 2003 Phylogeny of Amaranthaceae and Chenopodiaceae and the evolution of C_4_ photosynthesis. International Journal of Plant Sciences 164, 959–986

[CIT0043] Keeley JE Rundel PW 2003 Evolution of CAM and C_4_ carbon-concentrating mechanisms. International Journal of Plant Sciences 164, S55–S77

[CIT0044] Koch K Kennedy RA 1980 Characteristics of crassulacean acid metabolism in the succulent C_4_ dicot, *Portulaca oleracea* L. Plant Physiology 65, 193–197 16661159 10.1104/pp.65.2.193 PMC440296

[CIT0045] Koch K Kennedy RA 1982 Crassulacean acid metabolism in the succulent C_4_ dicot, *Portulaca oleracea* L. under natural environmental conditions. Plant Physiology 69, 757–761 16662291 10.1104/pp.69.4.757 PMC426300

[CIT0046] Kraybill AA Martin CE 1996 Crassulacean acid metabolism in three species of the C_4_ genus Portulaca. International Journal of Plant Sciences 157, 103–109

[CIT0047] Langmead B Salzberg S 2012 Fast gapped-read alignment with Bowtie 2. Nature Methods 9, 357–359 22388286 10.1038/nmeth.1923 PMC3322381

[CIT0048] Lara MV Chuong SDX Akhani H Andreo CS Edwards GE 2006 Species having C_4_ single-cell-type photosynthesis in the Chenopodiaceae family evolved a photosynthetic phosphoenolpyruvate carboxylase like that of Kranz-type C_4_ species. Plant Physiology 142, 673–684 16920871 10.1104/pp.106.085829 PMC1586054

[CIT0049] Lara MV Disante KB Podesta FE Andreo CS Drincovich MF 2003 Induction of a Crassulacean acid like metabolism in the C_4_ succulent plant, *Portulaca oleracea* L.: physiological and morphological changes are accompanied by specific modifications in phosphoenolpyruvate carboxylase. Photosynthesis Research 77, 241–254 16228379 10.1023/A:1025834120499

[CIT0050] Lara MV Drincovich MF Andrea CS 2004 Induction of a crassulacean acid-like metabolism in the C_4_ succulent plant, *Portulaca oleracea* L.: study of enzymes involved in carbon fixation and carbohydrate metabolism. Plant Cell and Environment 45, 618–626 10.1093/pcp/pch073 15169944

[CIT0051] Lepiniec L Vidal J Chollet R Gadal P Cretin C 1994 Phosphoenolpyruvate carboxylase: structure, function, regulation and evolution. Plant Science 99, 111–124

[CIT0052] Ludwig M 2011 The molecular evolution of β-carbonic anhydrase in *Flaveria* . Journal of Experimental Botany 62, 3071–3081 21406474 10.1093/jxb/err071

[CIT0053] Mazen AMA 1996 Changes in levels of phosphoenolpyurvate carboxylase with induction of Crassulacean acid metabolism (CAM)-like behavior in the C_4_ plant *Portulaca oleracea* . Physiologia Plantarum 98, 111–116

[CIT0054] Mazen AMA 2000 Changes in properties of phosphoenolpyruvate carboxylase with induction of Crassulacean Acid Metabolism (CAM) in the C_4_ plant *Portulaca olearacea* . Photosynthetica 38, 385–391

[CIT0055] Monson RK 2003 Gene duplication, neofunctionalization, and the evolution of C_4_ photosynthesis. International Journal of Plant Sciences 164, S43–S54

[CIT0056] Nelson EA Sage TL Sage RF 2005 Functional leaf anatomy of plants with crassulacean acid metabolism. Functional Plant Biology 32, 409–419 10.1071/FP04195 32689143

[CIT0057] Nimmo HG 2003 How to tell the time: the regulation of phosphoenolpyruvate carboxylase in Crassulacean acid metabolism (CAM) plants. Biochemical Society Transactions 31, 728–730 12773193 10.1042/bst0310728

[CIT0058] Nyffeler R Eggli U 2010 Disintegrating Portulacaceae: a new familial classification of the suborder Portulacineae (Caryophyllales) based on molecular and morphological data. Taxon 59, 227–240

[CIT0059] Nyffeler R Eggli U Ogburn M Edwards EJ 2008 Variations on a theme: repeated evolution of succulent life forms in the Portulacineae (Caryophyllales). Haseltonia 14, 26–36

[CIT0060] Ocampo G Columbus JT 2010 Molecular phylogenetics of suborder Cactineae (Caryophyllales), including insights into photosynthetic diversification and historical biogeography. American Journal of Botany 97, 1827–1847 21616822 10.3732/ajb.1000227

[CIT0061] Ocampo G Columbus JT 2012 Molecular phylogenetics, historical biogeography, and chromosome number evolution of *Portulaca* (Portulacaceae). Molecular Phylogenetics and Evolution 63, 97–112 22210411 10.1016/j.ympev.2011.12.017

[CIT0062] Ocampo G Koteyeva NK Voznesenskaya EV Edwards GE Sage TL Sage RF Columbus JT 2013 Evolution of leaf anatomy and photosynthetic pathways in Portulacaceae. American Journal of Botany 100, 2388–2402 24259525 10.3732/ajb.1300094

[CIT0063] Ogburn RM Edwards EJ 2013 Repeated origin of three-dimensional leaf venation releases constraints on the evolution of succulence in plants. Current Biology 23, 722–726 23583553 10.1016/j.cub.2013.03.029

[CIT0064] Osmond CB 1978 Crassulacean acid metabolism. A curiosity in context. Annual Reviews of Plant Physiology 29, 379–414

[CIT0065] Rambaut A Drummond AJ 2007 Tracer v1.4, available at http://beadt.bio.ed.ac.uk/Tracer

[CIT0066] Rao SK Magnin NC Reiskind JB Bowes G 2002 Photosynthetic and other phosphoenolpyruvate carboxylase isoforms in the single-cell, facultative C_4_ system of *Hydrilla verticillata* . Plant Physiology 130, 876–886 12376652 10.1104/pp.008045 PMC166614

[CIT0067] Rao SK Reiskind JB Bowes G 2008 Kinetic analyses of recombinant isoforms of phosphoenolpyruvate carboxylase from *Hydrilla verticillata* leaves and the impact of substituting a C_4_-signature serine. Plant Science 174, 475–483

[CIT0068] Raven JA Cockell CS De La Rocha CS 2008 The evolution of inorganic carbon concentrating mechanisms in photosynthesis. Philosophical Transactions of the Royal Society of London Series B 363, 2641–2650 18487130 10.1098/rstb.2008.0020 PMC2606764

[CIT0069] Rickers J Cushman JC Michalowski CB Schmitt JM Bohnert HJ 1989 Expression of the CAM-form of phospho(enol)pyruvate carboxylase and nucleotide sequence of a full length cDNA of *Mesembryanthemum crystallinum* . Molecular and General Genetics 215, 447–454 2710107 10.1007/BF00427042

[CIT0070] Ronquist F Huelsenbeck JP 2003 MrBayes 3: Bayesian phylogenetic inference under mixed models. Bioinformatics 19, 1572–1574 12912839 10.1093/bioinformatics/btg180

[CIT0071] Sage RF 2001 Environmental and evolutionary preconditions for the origin and diversification of the C_4_ photosynthetic syndrome. Plant Biology 3, 202–213

[CIT0072] Sage RF 2002 Are crassulacean acid metabolism and C_4_ photosynthesis incompatible? Functional Plant Biology 29, 775–785 10.1071/PP01217 32689525

[CIT0073] Sage RF Christin PA Edwards EJ 2011 The C_4_ plant lineages of planet Earth. Journal of Experimental Botany 62, 3155–3169 21414957 10.1093/jxb/err048

[CIT0074] Sage RF Sage TL Kocacinar F 2012 Photorespiration and the evolution of C_4_ photosynthesis. Annual Reviews of Plant Biology 63, 19–47 10.1146/annurev-arplant-042811-105511 22404472

[CIT0075] Silvera K Santiago LS Winter K 2005 Distribution of crassulacean acid metabolism in orchids of Panama: evidence of selection for weak and strong modes. Functional Plant Biology 32, 397–407 10.1071/FP04179 32689142

[CIT0076] Soltis DE Smith SA Cellinese N 2011 Angiosperm phylogeny: 17 genes, 640 taxa. American Journal of Botany 98, 704–730 21613169 10.3732/ajb.1000404

[CIT0077] Svensson P Bläsing OE Westhoff P 2003 Evolution of C_4_ phosphoenolpyruvate carboxylase. Archives of Biochemistry and Biophysics 414, 180–188 12781769 10.1016/s0003-9861(03)00165-6

[CIT0078] Tausta SL Miller Coyle H Rothermel B Stifel V Nelson T 2002 Maize C_4_ and non-C_4_ NADP-dependent malic enzymes are encoded by distinct genes derived from a plastid-localized ancestor. Plant Molecular Biology 50, 635–652 12374297 10.1023/a:1019998905615

[CIT0079] Thompson JD Higgins DJ Gibson TJ 1994 ClustalW: improving the sensitivity of progressive multiple sequence alignment through sequence weighting, position specific gap penalties and matrix choice. Nucleic Acids Research 22, 4673–4680 7984417 10.1093/nar/22.22.4673 PMC308517

[CIT0080] Voznesenskaya EV Koteyava NK Edwards GE Ocampo G 2010 Revealing diversity in structural and biochemical forms of C_4_ photosynthesis in genus *Portulaca* L. (Portulacaceae). Journal of Experimental Botany 61, 3647–3662 20591900 10.1093/jxb/erq178 PMC2921202

[CIT0081] Yang ZH 2007 PAML4: phylogenetic analysis by maximum likelihood. Molecular Biology and Evolution 24, 1586–1591 17483113 10.1093/molbev/msm088

[CIT0082] Yang ZH Nielsen R 2002 Codon-substitutions models for detecting molecular adaptation at individual sites along specific lineages. Molecular Biology and Evolution 19, 908–917 12032247 10.1093/oxfordjournals.molbev.a004148

