# Supplementary material for: Shared origins of a key enzyme during the evolution of C4 and CAM metabolism
Source: J Exp Bot. 2014 Mar 17;65(13):3609–21. doi: 10.1093/jxb/eru087 (PMC4085957; doi:10.1093/jxb/eru087)
Supplement: Supplementary Data [file supp_65_13_3609__index.html]

Shared origins of a key enzyme during the evolution of C4 and CAM metabolism — Supplementary Data 

# Shared origins of a key enzyme during the evolution of C4 and CAM metabolism

## Supplementary Data

Data files

**Files in this Data Supplement:**

- Supplementary Data - Supplementary Data
