# Supplementary material for: Shared origins of a key enzyme during the evolution of C4 and CAM metabolism
Source: J Exp Bot. 2014 Mar 17;65(13):3609–21. doi: 10.1093/jxb/eru087 (PMC4085957; doi:10.1093/jxb/eru087)
Supplement: Supplementary Data [file supp_eru087_eru087_SI.pdf]

Supplementary Material for:

Shared origins of a key enzyme during the evolution of C_4 and CAM metabolism

Pascal-Antoine Christin, Monica Arakaki, Colin P. Osborne, Andrea Bräutigam, Rowan F. Sage, Julian M. Hibberd, Steven Kelly, Sarah Covshoff, Gane Ka-Shu Wong, Lillian Hancock, Erika J. Edwards

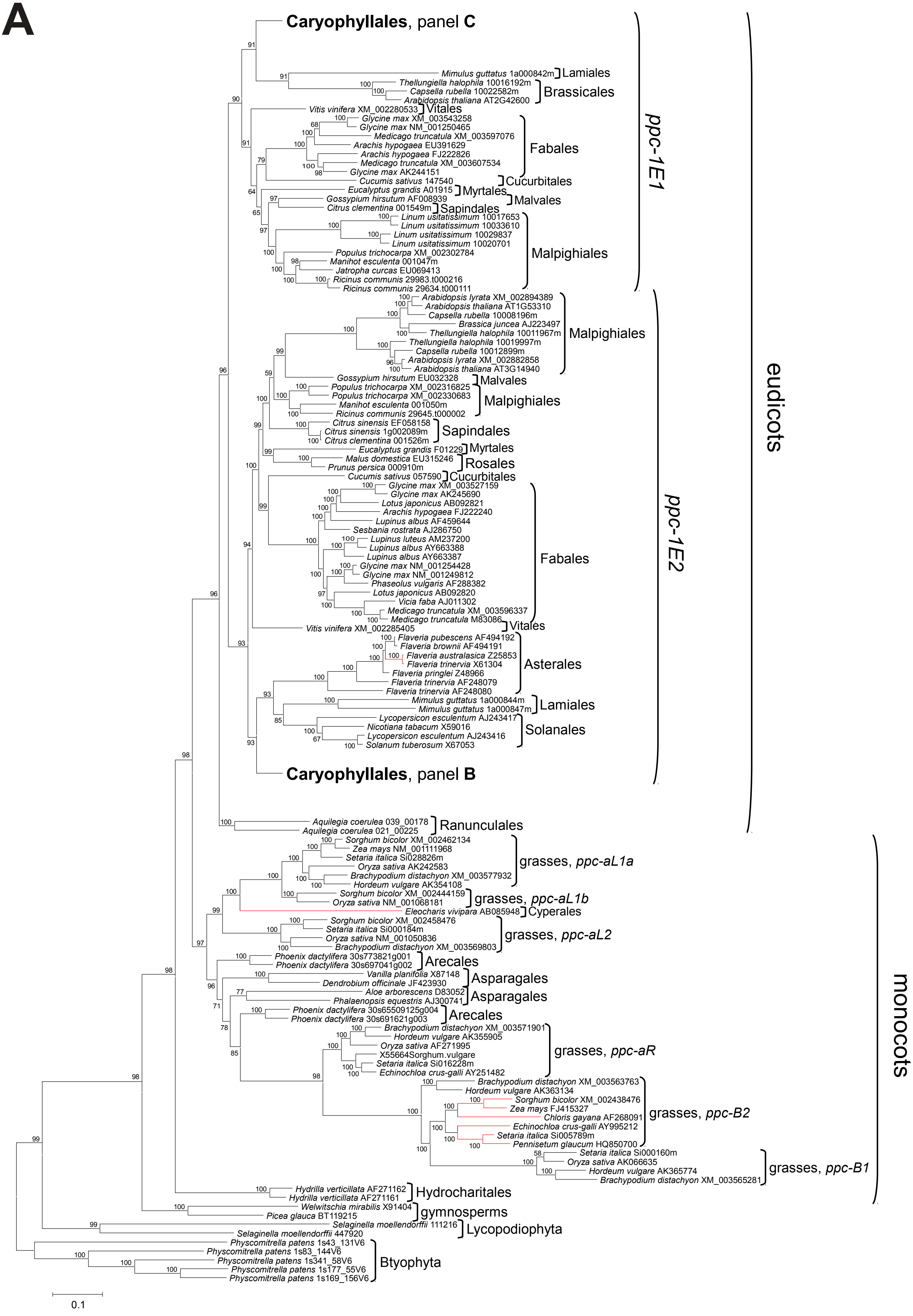
A
Caryophyllales, panel C
Mimulus guttatus 1a000842m
Lamiales
Thellungiella halophila 10016192m
Capsella rubella 10022582m
Arabidopsis thaliana AT2G42600
Brassicales
Vitis vinifera XM_002280533
Vitales
Glycine max XM_003543258
Glycine max NM_001250465
Medicago truncatula XM_003597076
Arachis hypogaea EU391629
Arachis hypogaea FJ222826
Medicago truncatula XM_003607534
Glycine max AK244151
Fabales
Cucumis sativus 147540
Cucurbitales
Eucalyptus grandis A01915
Myrtales
Gossypium hirsutum AF008939
Malvales
Citrus clementina 001549m
Sapindales
Linum usitatissimum 10017653
Linum usitatissimum 10033610
Linum usitatissimum 10029837
Linum usitatissimum 10020701
Populus trichocarpa XM_002302784
Manihot esculenta 001047m
Jatropha curcas EU069413
Ricinus communis 29983.t000216
Ricinus communis 29634.t000111
Malpighiales
ppc-1E1
Arabidopsis lyrata XM_002894389
Arabidopsis thaliana AT1G53310
Capsella rubella 10008196m
Brassica juncea AJ223497
Thellungiella halophila 10011967m
Thellungiella halophila 10019997m
Capsella rubella 10012899m
Arabidopsis lyrata XM_002882858
Arabidopsis thaliana AT3G14940
Malpighiales
Gossypium hirsutum EU032328
Malvales
Populus trichocarpa XM_002316825
Populus trichocarpa XM_002330683
Manihot esculenta 001050m
Ricinus communis 29645.t000002
Malpighiales
Citrus sinensis EF058158
Citrus sinensis 1g002089m
Citrus clementina 001526m
Sapindales
Eucalyptus grandis F01229
Myrtales
Malus domestica EU315246
Prunus persica 000910m
Rosales
Cucumis sativus 057590
Cucurbitales
Glycine max XM_003527159
Glycine max AK245690
Lotus japonicus AB092821
Arachis hypogaea FJ222240
Lupinus albus AF459644
Sesbania rostrata AJ286750
Lupinus luteus AM237200
Lupinus albus AY663388
Lupinus albus AY663387
Glycine max NM_001254428
Glycine max NM_001249812
Phaseolus vulgaris AF288382
Lotus japonicus AB092820
Vicia faba AJ011302
Medicago truncatula XM_003596337
Medicago truncatula M83086
Fabales
Vitis vinifera XM_002285405
Vitales
Flaveria pubescens AF494192
Flaveria brownii AF494191
Flaveria australasica Z25853
Flaveria trinervia X61304
Flaveria pringlei Z48966
Flaveria trinervia AF248079
Flaveria trinervia AF248080
Asterales
Mimulus guttatus 1a000844m
Mimulus guttatus 1a000847m
Lamiales
Lycopersicon esculentum AJ243417
Nicotiana tabacum X59016
Lycopersicon esculentum AJ243416
Solanum tuberosum X67053
Solanales
Caryophyllales, panel B
ppc-1E2
eudicots
Aquilegia coerulea 039_00178
Aquilegia coerulea 021_00225
Ranunculales
Sorghum bicolor XM_002462134
Zea mays NM_001111968
Setaria italica Si028826m
Oryza sativa AK242583
Brachypodium distachyon XM_003577932
Hordeum vulgare AK354108
grasses, ppc-aL1a
Sorghum bicolor XM_002444159
Oryza sativa NM_001068181
grasses, ppc-aL1b
Eleocharis vivipara AB085948
Cyperales
Sorghum bicolor XM_002458476
Setaria italica Si000184m
Oryza sativa NM_001050836
Brachypodium distachyon XM_003569803
grasses, ppc-aL2
Phoenix dactylifera 30s773821g001
Phoenix dactylifera 30s697041g002
Arecales
Vanilla planifolia X87148
Dendrobium officinale JF423930
Asparagales
Aloe arborescens D83052
Phalaenopsis equestris AJ300741
Asparagales
Phoenix dactylifera 30s65509125g004
Phoenix dactylifera 30s691621g003
Arecales
Brachypodium distachyon XM_003571901
Hordeum vulgare AK355905
Oryza sativa AF271995
X55664Sorghum.vulgare
Setaria italica Si016228m
Echinochloa crus-galli AY251482
grasses, ppc-aR
Brachypodium distachyon XM_003563763
Hordeum vulgare AK363134
Sorghum bicolor XM_002438476
Zea mays FJ415327
Chloris gayana AF268091
Echinochloa crus-galli AY995212
Setaria italica Si005789m
Pennisetum glaucum HQ850700
grasses, ppc-B2
Setaria italica Si000160m
Oryza sativa AK066635
Hordeum vulgare AK365774
Brachypodium distachyon XM_003565281
grasses, ppc-B1
monocots
Hydrilla verticillata AF271162
Hydrilla verticillata AF271161
Hydrocharitales
Welwitschia mirabilis X91404
Picea glauca BT119215
gymnosperms
Selaginella moellendorffii 111216
Selaginella moellendorffii 447920
Lycopodiophyta
Physcomitrella patens 1s43_131V6
Physcomitrella patens 1s83_144V6
Physcomitrella patens 1s341_58V6
Physcomitrella patens 1s177_55V6
Physcomitrella patens 1s169_156V6
Btyophyta
0.1

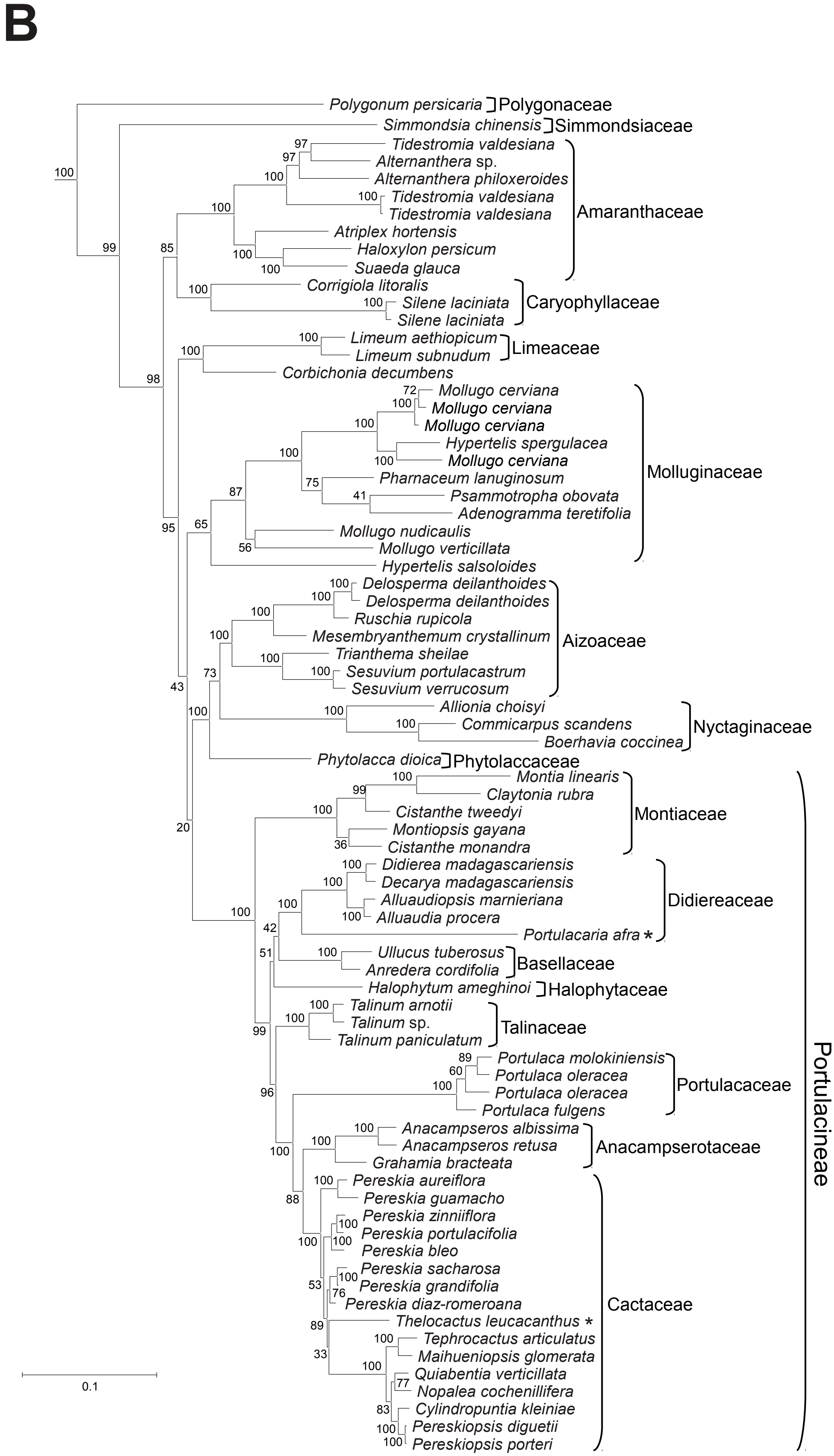
B
Polygonum persicaria
Polygonaceae
Simmondsia chinensis
Simmondsiaceae
Tidestromia valdesiana
Alternanthera sp.
Alternanthera philoxeroides
Tidestromia valdesiana
Tidestromia valdesiana
Atriplex hortensis
Haloxylon persicum
Suaeda glauca
Amaranthaceae
Corrigiola litoralis
Silene laciniata
Silene laciniata
Caryophyllaceae
Limeum aethiopicum
Limeum subnudum
Limeaceae
Corbichonia decumbens
Mollugo cerviana
Mollugo cerviana
Mollugo cerviana
Hypertelis spergulacea
Mollugo cerviana
Pharnaceum lanuginosum
Psammotropha obovata
Adenogramma teretifolia
Mollugo nudicaulis
Mollugo verticillata
Molluginaceae
Hypertelis salsoloides
Delosperma deilanthoides
Delosperma deilanthoides
Ruschia rupicola
Mesembryanthemum crystallinum
Trianthema sheilae
Sesuvium portulacastrum
Sesuvium verrucosum
Aizoaceae
Allionia choisyi
Commicarpus scandens
Boerhavia coccinea
Nyctaginaceae
Phytolacca dioica
Phytolaccaceae
Montia linearis
Claytonia rubra
Cistanthe tweedyi
Montiopsis gayana
Cistanthe monandra
Montiaceae
Didierea madagascariensis
Decarya madagascariensis
Alluaudiopsis marnieriana
Alluaudia procera
Portulacaria afra *
Didiereaceae
Ullucus tuberosus
Anredera cordifolia
Basellaceae
Halophytum ameghinoi
Halophytaceae
Talinum arnotii
Talinum sp.
Talinum paniculatum
Talinaceae
Portulaca molokiniensis
Portulaca oleracea
Portulaca oleracea
Portulaca fulgens
Portulacaceae
Anacampseros albissima
Anacampseros retusa
Grahamia bracteata
Anacampserotaceae
Pereskia aureiflora
Pereskia guamacho
Pereskia zinniiflora
Pereskia portulacifolia
Pereskia bleo
Pereskia sacharosa
Pereskia grandifolia
Pereskia diaz-romeroana
Thelocactus leucacanthus *
Tephrocactus articulatus
Maihueniopsis glomerata
Quiabentia verticillata
Nopalea cochenillifera
Cylindropuntia kleiniae
Pereskiopsis diguetii
Pereskiopsis porteri
Cactaceae
Portulacineae
0.1

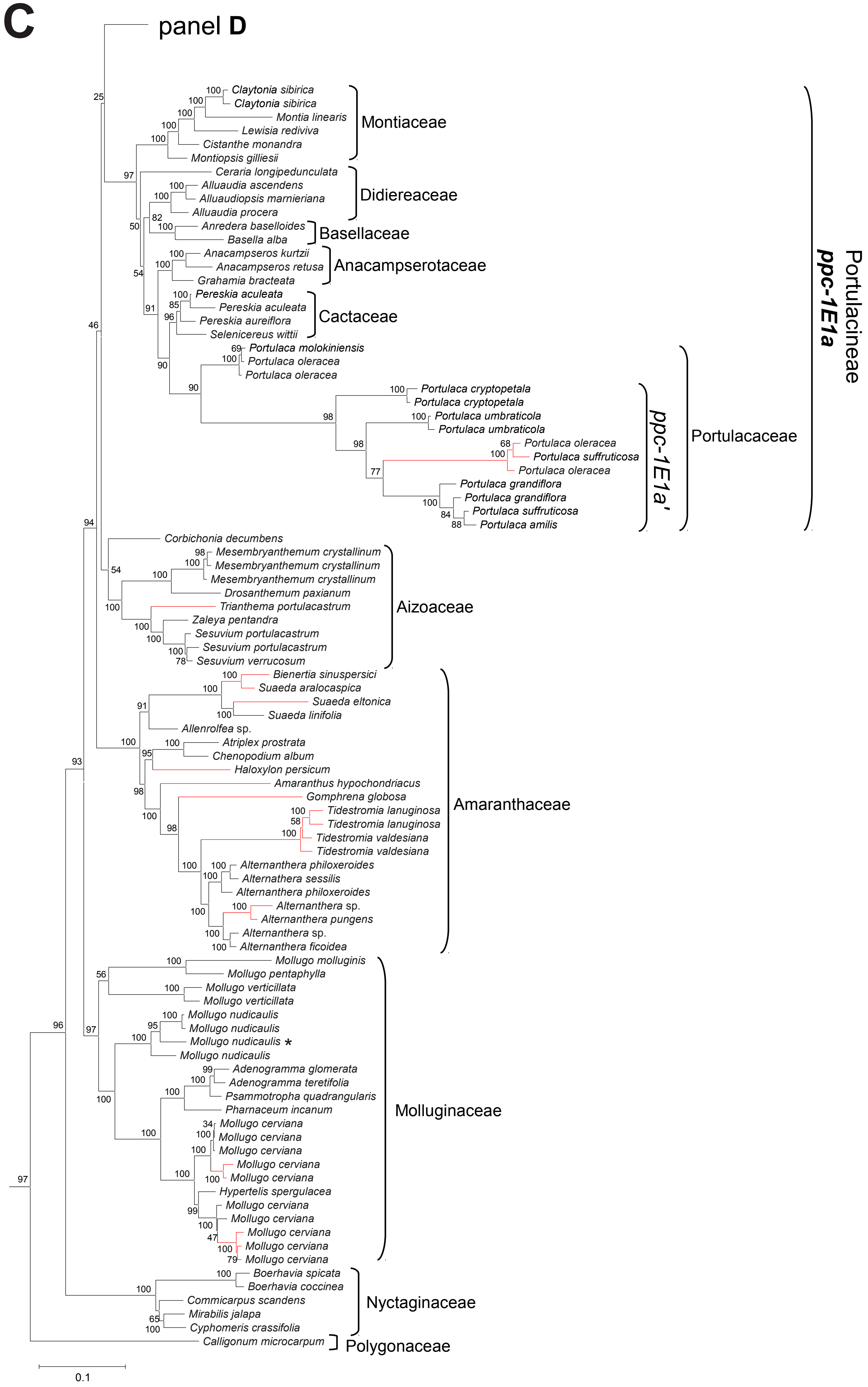

C
panel D
Claytonia sibirica
Claytonia sibirica
Montia linearis
Lewisia rediviva
Cistanthe monandra
Montiopsis gilliesii
Montiaceae
Ceraria longipedunculata
Alluaudia ascendens
Alluaudiopsis marnieriana
Alluaudia procera
Didiereaceae
Anredera baselloides
Basella alba
Basellaceae
Anacampseros kurtzii
Anacampseros retusa
Grahamia bracteata
Anacampserotaceae
Pereskia aculeata
Pereskia aculeata
Pereskia aureiflora
Selenicereus wittii
Cactaceae
Portulaca molokiniensis
Portulaca oleracea
Portulaca oleracea
Portulaca cryptopetala
Portulaca cryptopetala
Portulaca umbraticola
Portulaca umbraticola
Portulaca oleracea
Portulaca suffruticosa
Portulaca oleracea
Portulaca grandiflora
Portulaca grandiflora
Portulaca suffruticosa
Portulaca amilis
ppc-1E1a'
Portulacaceae
Portulacineae
ppc-1E1a
Corbichonia decumbens
Mesembryanthemum crystallinum
Mesembryanthemum crystallinum
Mesembryanthemum crystallinum
Drosanthemum paxianum
Trianthema portulacastrum
Zaleya pentandra
Sesuvium portulacastrum
Sesuvium portulacastrum
Sesuvium verrucosum
Aizoaceae
Bienertia sinuspersici
Suaeda aralocaspica
Suaeda eltonica
Suaeda linifolia
Allenrolfea sp.
Atriplex prostrata
Chenopodium album
Haloxylon persicum
Amaranthus hypochondriacus
Gomphrena globosa
Tidestromia lanuginosa
Tidestromia lanuginosa
Tidestromia valdesiana
Tidestromia valdesiana
Alternanthera philoxeroides
Alternathera sessilis
Alternanthera philoxeroides
Alternanthera sp.
Alternanthera pungens
Alternanthera sp.
Alternanthera ficoidea
Amaranthaceae
Mollugo molluginis
Mollugo pentaphylla
Mollugo verticillata
Mollugo verticillata
Mollugo nudicaulis
Mollugo nudicaulis
Mollugo nudicaulis *
Mollugo nudicaulis
Adenogramma glomerata
Adenogramma teretifolia
Psammotropha quadrangularis
Pharnaceum incanum
Mollugo cerviana
Mollugo cerviana
Mollugo cerviana
Mollugo cerviana
Mollugo cerviana
Hypertelis spergulacea
Mollugo cerviana
Mollugo cerviana
Mollugo cerviana
Mollugo cerviana
Mollugo cerviana
Molluginaceae
Boerhavia spicata
Boerhavia coccinea
Commicarpus scandens
Mirabilis jalapa
Cyphomeris crassifolia
Nyctaginaceae
Calligonum microcarpum
Polygonaceae
0.1

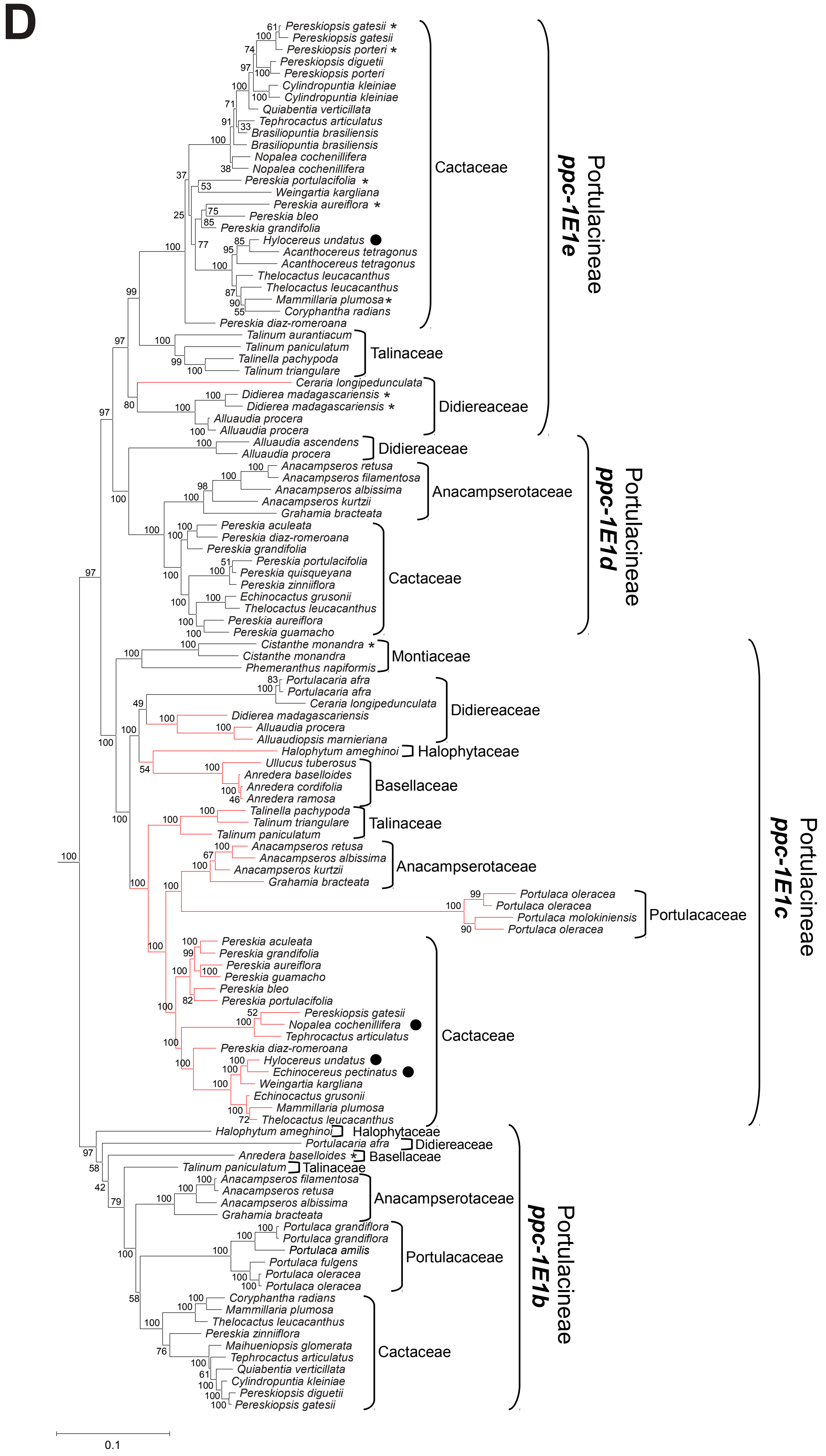
D
Pereskiopsis gatesii *
Pereskiopsis gatesii
Pereskiopsis porteri *
Pereskiopsis diguetii
Pereskiopsis porteri
Cylindropuntia kleiniae
Cylindropuntia kleiniae
Quiabentia verticillata
Tephrocactus articulatus
Brasiliopuntia brasiliensis
Brasiliopuntia brasiliensis
Nopalea cochenillifera
Nopalea cochenillifera
Pereskia portulacifolia *
Weingartia kargliana
Pereskia aureiflora *
Pereskia bleo
Pereskia grandifolia
Hylocereus undatus
Acanthocereus tetragonus
Acanthocereus tetragonus
Thelocactus leucacanthus
Thelocactus leucacanthus
Mammillaria plumosa *
Coryphantha radians
Pereskia diaz-romeroana
Cactaceae
Talinum aurantiacum
Talinum paniculatum
Talinella pachypoda
Talinum triangulare
Talinaceae
Ceraria longipedunculata
Didierea madagascariensis *
Didierea madagascariensis *
Alluaudia procera
Alluaudia procera
Didiereaceae
Portulacineae
ppc-1E1e
Alluaudia ascendens
Alluaudia procera
Didiereaceae
Anacampseros retusa
Anacampseros filamentosa
Anacampseros albissima
Anacampseros kurtzii
Grahamia bracteata
Anacampserotaceae
Pereskia aculeata
Pereskia diaz-romeroana
Pereskia grandifolia
Pereskia portulacifolia
Pereskia quisqueyana
Pereskia zinniiflora
Echinocactus grusonii
Thelocactus leucacanthus
Pereskia aureiflora
Pereskia guamacho
Cactaceae
Portulacineae
ppc-1E1d
Cistanthe monandra *
Cistanthe monandra
Phemeranthus napiformis
Montiaceae
Portulacaria afra
Portulacaria afra
Ceraria longipedunculata
Didierea madagascariensis
Alluaudia procera
Alluaudiopsis marnieriana
Didiereaceae
Halophytum ameghinoi
Halophytaceae
Ullucus tuberosus
Anredera baselloides
Anredera cordifolia
Anredera ramosa
Basellaceae
Talinella pachypoda
Talinum triangulare
Talinum paniculatum
Talinaceae
Anacampseros retusa
Anacampseros albissima
Anacampseros kurtzii
Grahamia bracteata
Anacampserotaceae
Portulaca oleracea
Portulaca oleracea
Portulaca molokiniensis
Portulaca oleracea
Portulacaceae
Pereskia aculeata
Pereskia grandifolia
Pereskia aureiflora
Pereskia guamacho
Pereskia bleo
Pereskia portulacifolia
Pereskiopsis gatesii
Nopalea cochenillifera
Tephrocactus articulatus
Pereskia diaz-romeroana
Hylocereus undatus
Echinocereus pectinatus
Weingartia kargliana
Echinocactus grusonii
Mammillaria plumosa
Thelocactus leucacanthus
Cactaceae
Portulacineae
ppc-1E1c
Halophytum ameghinoi
Halophytaceae
Portulacaria afra
Didiereaceae
Anredera baselloides *
Basellaceae
Talinum paniculatum
Talinaceae
Anacampseros filamentosa
Anacampseros retusa
Anacampseros albissima
Grahamia bracteata
Anacampserotaceae
Portulaca grandiflora
Portulaca grandiflora
Portulaca amilis
Portulaca fulgens
Portulaca oleracea
Portulaca oleracea
Portulacaceae
Coryphantha radians
Mammillaria plumosa
Thelocactus leucacanthus
Pereskia zinniiflora
Maihueniopsis glomerata
Tephrocactus articulatus
Quiabentia verticillata
Cylindropuntia kleiniae
Pereskiopsis diguetii
Pereskiopsis gatesii
Cactaceae
Portulacineae
ppc-1E1b
0.1

Supplementary Figure 1: Phylogenetic relationships among *ppc-1* genes.

This phylogenetic tree was obtained through Bayesian inference on nucleotide sequences. Names of taxonomic groups and gene lineages are indicated on the right. Branches in lineages presenting a Ser780 are highlighted in red. Bayesian support values are indicated near branches. Asterisks indicate putative pseudogenes with one or several stop codons in the coding sequence. Black circles indicate sequences that were isolated from cacti cDNA. **A** Complete phylogenetic tree, **B** *ppc-1E2* of Caryophyllales, **C** and **D** *ppc-1E1* of Caryophyllales.

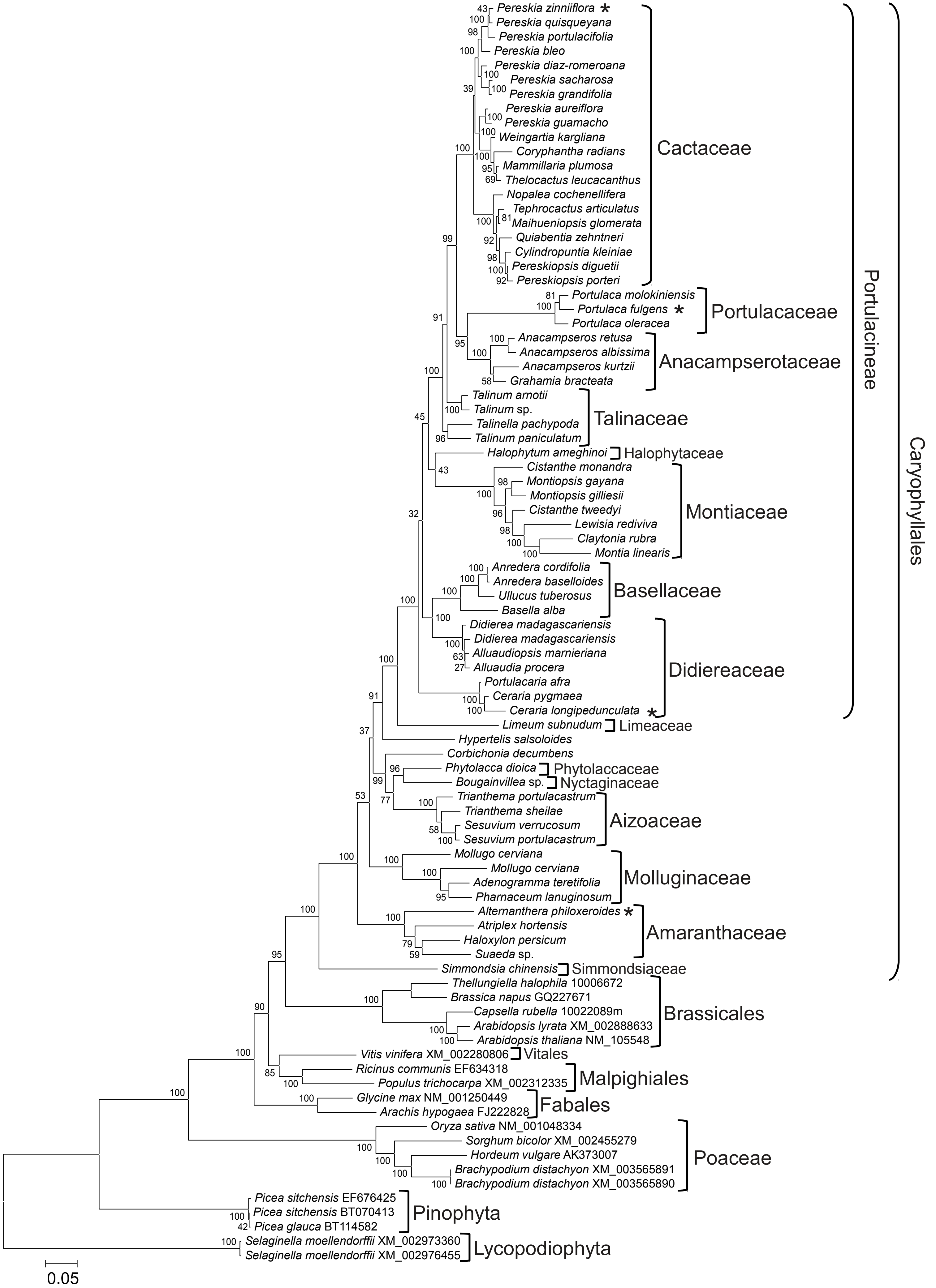

Pereskia zinniiflora *
Pereskia quisqueyana
Pereskia portulacifolia
Pereskia bleo
Pereskia diaz-romeroana
Pereskia sacharosa
Pereskia grandifolia
Pereskia aureiflora
Pereskia guamacho
Weingartia kargliana
Coryphantha radians
Mammillaria plumosa
Thelocactus leucacanthus
Nopalea cochenellifera
Tephrocactus articulatus
Maihueniopsis glomerata
Quiabentia zehntneri
Cylindropuntia kleiniae
Pereskiopsis diguetii
Pereskiopsis porteri
Cactaceae
Portulaca molokiniensis
Portulaca fulgens *
Portulaca oleracea
Portulacaceae
Anacampseros retusa
Anacampseros albissima
Anacampseros kurtzii
Grahamia bracteata
Anacampserotaceae
Talinum arnotii
Talinum sp.
Talinella pachypoda
Talinum paniculatum
Talinaceae
Halophytum ameghinoi
Halophytaceae
Cistanthe monandra
Montiopsis gayana
Montiopsis gilliesii
Cistanthe tweedyi
Lewisia rediviva
Claytonia rubra
Montia linearis
Montiaceae
Anredera cordifolia
Anredera baselloides
Ullucus tuberosus
Basella alba
Basellaceae
Didierea madagascariensis
Didierea madagascariensis
Alluaudiopsis marnieriana
Alluaudia procera
Portulacaria afra
Ceraria pygmaea
Ceraria longipedunculata *
Didiereaceae
Limeum subnudum
Limeaceae
Hypertelis salsoloides
Corbichonia decumbens
Phytolacca dioica
Phytolaccaceae
Bougainvillea sp.
Nyctaginaceae
Trianthema portulacastrum
Trianthema sheilae
Sesuvium verrucosum
Sesuvium portulacastrum
Aizoaceae
Mollugo cerviana
Mollugo cerviana
Adenogramma teretifolia
Pharnaceum lanuginosum
Molluginaceae
Alternanthera philoxeroides *
Atriplex hortensis
Haloxylon persicum
Suaeda sp.
Amaranthaceae
Simmondsia chinensis
Simmondsiaceae
Thellungiella halophila 10006672
Brassica napus GQ227671
Capsella rubella 10022089m
Arabidopsis lyrata XM_002888633
Arabidopsis thaliana NM_105548
Brassicales
Vitis vinifera XM_002280806
Vitales
Ricinus communis EF634318
Populus trichocarpa XM_002312335
Malpighiales
Glycine max NM_001250449
Arachis hypogaea FJ222828
Fabales
Oryza sativa NM_001048334
Sorghum bicolor XM_002455279
Hordeum vulgare AK373007
Brachypodium distachyon XM_003565891
Brachypodium distachyon XM_003565890
Poaceae
Picea sitchensis EF676425
Picea sitchensis BT070413
Picea glauca BT114582
Pinophyta
Selaginella moellendorffii XM_002973360
Selaginella moellendorffii XM_002976455
Lycopodiophyta
Portulacineae
Caryophyllales
0.05

Supplementary Figure 2: Phylogenetic relationships among *ppc-2* genes.

This phylogenetic tree was obtained through Bayesian inference on nucleotide sequences. Names of taxonomic groups are indicated on the right. Bayesian support values are indicated near branches.

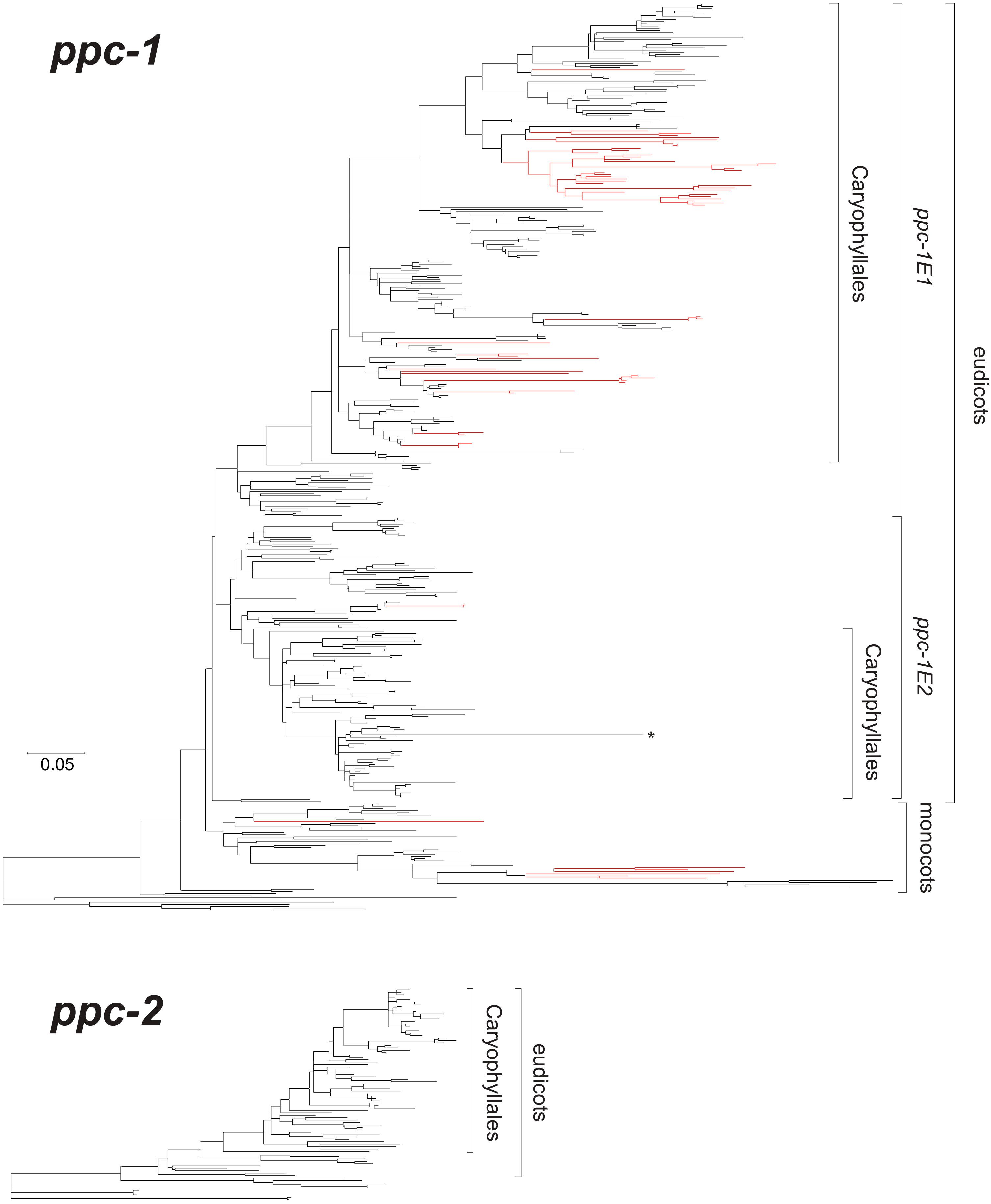
ppc-1
Caryophyllales
ppc-1E1
eudicots
Caryophyllales
ppc-1E2
monocots
*
0.05
ppc-2
Caryophyllales
eudicots

Supplementary Figure 3: Amino acid changes on genes encoding PEPC.

The topology was inferred on nucleotide sequences, but branch lengths were estimated based on amino acid sequences. The branch lengths inferred on nucleotide sequences, together with all species names and support values, are available in Figures S1 and S2. The names of the main groups are indicated on the right. Groups of genes containing a Ser780 are highlighted by red branches. The asterisk highlights a pseudogene with multiple stop codons.

Supplementary Table 1: Sample of Caryophyllales (excluding Portulacineae) used for analyses of PEPC encoding genes.

Species	Voucher	*ppc-1E1*	*ppc-1E2*	*ppc-2*
**Simmondsiaceae**				
Simmondsia chinensis (Link) C.K. Schneid.	Clement 235 (YU)	-	KJ161696	KJ161762
**Polygonaceae**				
Calligonum microcarpum I.G. Borshch.	Sage 0509-17	KJ161675	-	-
Polygonum persicaria L.	-	-	KJ161697	-
**Caryophyllaceae**				
Corrigiola litoralis L.	FR-0082024 (FR)	-	FN868276	-
Silene laciniata Cav.	Clement 236 (Y)	-	KJ161698, KJ161699	-
**Amaranthaceae**				
Allenrolfea sp.	Christin s.n. (BRU)	KJ161676	-	-
Alternanthera ficoidea (L.) P. Beauv.	-	AY950666	-	-
Alternanthera philoxeroides (Mart.) Griseb.	Sage 0509-13	KJ161677, KJ161678	KJ161700	KJ161763
Alternanthera pungens Kunth	-	AY950665	-	-
Alternathera sessilis (L.) R. Br.	-	AY950667	-	-
Alternanthera sp.	Sage 0509-16	KJ161679	KJ161701	-
Amaranthus hypochondriacus L.	-	Z68125	-	-
Atriplex hortensis L.	-	-	KJ161702	KJ161764
Atriplex prostrata Boucher ex DC.	-	KJ161680	-	-
Bienertia sinuspersici Akhani	-	DQ538352	-	-
Chenopodium album L.	-	KJ161681	-	-
Gomphrena globosa L.	-	KJ161682	-	-
Haloxylon persicum Bunge ex Boiss. & Buhse	Sage 0509-H8	KJ161683	KJ161703	KJ161765
Suaeda aralocaspica Freitag & Schutze	-	DQ538353	-	-
Suaeda eltonica Iljin	-	DQ538354	-	-
Suaeda glauca (Bunge) Bunge	-	-	EU629170	-
Suaeda linifolia Pall.	-	DQ538355	-	-
Suaeda sp.	Christin s.n. (BRU)	-	-	KJ161766
Tidestromia lanuginosa (Nutt.) Standl.	Higgens et al. 21768 (NY)	KJ161684, KJ161685	-	-
Tidestromia valdesiana Sánchez-del Pino & Flores Olv.	Sanchez del Pino (NY)	KJ161686, KJ161687	KJ161704, KJ161705, KJ161706	-
**Aizoaceae**				
Delosperma deilanthoides S.A. Hammer	998369/0 (ZSS)	-	KJ161707, KJ161708	-
Drosanthemum paxianum Schwantes	-	Y17844	-	-
Mesembryanthemum crystallinum L.	-	X13660, AJ312642, AJ312643	-	-
Ruschia rupicola (Engl.) Schwante*s*	Brack 1508 (ZSS)	-	KJ161709	-
Sesuvium portulacastrum (L.) L.	-	KJ161688	KJ161710	KJ161767
Sesuvium verrucosum Raf.	Sage 0509-06	-	KJ161711	KJ161768
Trianthema portulacastrum L.	Christin 163 (BRU)	KJ161689	-	KJ161769
Trianthema sheilae A.G.Mill. & J.A.Nyberg	-	-	KJ161712	KJ161770
Zaleya pentandra (L.) C. Jeffrey	-	KJ161690	-	-

**Incertae sedis**				
Corbichonia decumbens (Forssk.) Exell	-	FN868270	FN868271	KJ161771
Hypertelis salsoloides (Burch.) Adamson	Ogburn 143 (BRU)	-	KJ161713	KJ161772
**Nyctaginaceae**				
Allionia choisyi Standl.	Douglas 2187 (DUKE)	-	KJ161714	-
Boerhavia coccinea Mill.	Douglas 2056 (NY)	KJ161691	KJ161715	-
Boerhavia spicata Choisy	Douglas 2171 (NY)	KJ161692	-	-
Bougainvillea sp.	Cultivated, Botanical Garden Zürich	-	-	KJ161773
Commicarpus scandens (L.) Standl.	-	KJ161693	KJ161716	-
Cyphomeris crassifolia (Standl.) Standl.	Douglas 2203	KJ161694	-	-
Mirabilis jalapa L.	-	KJ161695	-	-
**Phytolaccaceae**				
Phytolacca dioica L.	871040/0 (ZSS)	-	KJ161717	KJ161774
**Limeaceae**				
Limeum aethiopicum Burm.f.		-	FN868277	-
Limeum subnudum Friedrich	Ogburn 154 (BRU)	-	KJ161718	KJ161775
**Molluginaceae**				
Adenogramma glomerata Druce	Ogburn 142 (BRU)	FN868266	-	-
Adenogramma teretifolia Adamson	Ogburn 156 (BRU)	FN868267	KJ161719	KJ161776
Hypertelis spergulacea E. Mey. ex Fenzl	Giess et al. 5366 (K)	FN868290	FN868289	-
Mollugo cerviana (L.) Ser.	Atwood & Welsh 10684 (NY)	FN868297, FN868296	KJ161720	KJ161777
Mollugo cerviana (L.) Ser.	Shekhawat 2008	FN868294, FN868295	-	-
Mollugo cerviana (L.) Ser.	Thulin et al. 11211 (UPS)	FN868298, FN868299	-	-
Mollugo cerviana (L.) Ser.	Potgieter 225 (K)	FN868301	FN868287	-
Mollugo cerviana (L.) Ser.	J. Sánchez Sánchez s.n. (G)	FN868300	FN868288	KJ161778
Mollugo cerviana (L.) Ser.	Lazarides & Palmer 243 (CANB)	FN868291	FN868285	-
Mollugo cerviana (L.) Ser.	G. Leach 2008 (CANB)	FN868292	-	-
Mollugo cerviana (L.) Ser.	Smyth 213 (CANB)	FN868293	-	-
Mollugo molluginis (F.Muell.) Druce	Telford 11746 (CANB)	FN868275	-	-
Mollugo nudicaulis Lam.	Liogier 16585 (NY)	FN868284	-	-
Mollugo nudicaulis Lam.	Devi Sept 1961 (CANB)	FN868281, FN868280	FN868282	-
Mollugo nudicaulis Lam.	Thulin & Bashir-Mohamed 6759 (UPS)	FN868283	-	-
Mollugo pentaphylla L.	Nee 42741 (NY)	FN868272	-	-
Mollugo verticillata L.	Sage & Sage 8-2007 (TRT)	FN868273	KJ161721	-
Mollugo verticillata L.	M. Nee 37372 (G)	FN868274	-	-
Pharnaceum incanum L.	Ogburn 148 (BRU)	FN868268	-	-
Pharnaceum lanuginosum J.C. Manning & Goldblatt	Ogburn 161 (BRU)	-	KJ161722	KJ161779
Psammotropha obovata Adamson	Hilliard & Burtt 7095 (K)	-	FN868278	-
Psammotropha quadrangularis (L. f.) Fenzl	Ogburn 160 (BRU)	FN868269	-	-

Supplementary Table 2: List of Portulacineae genes encoding PEPC analysed

Species	Voucher	*ppc-1E1a*	*ppc-1E1b*	*ppc-1E1c*	*ppc-1E1d*	*ppc-1E1e*	*ppc-1E2*	*ppc-2*
**Montiaceae**								
Cistanthe monandra (Nutt.) Hershkovitz	Ogburn 137 (BRU)	KJ161552	-	KJ161591, KJ161592	-	-	KJ161723	KJ161780
Cistanthe tweedyi (A. Gray) Hersk.	Ogburn 182 (BRU)	-	-	-	-	-	KJ161724	KJ161781
Claytonia rubra (Howell) Tidestr.	Ogburn 183 (BRU)	-	-	-	-	-	KJ161725	KJ161782
Lewisia rediviva Pursh	Ogburn 185 (BRU)	KJ161553	-	-	-	-	-	KJ161783
Montia linearis (Douglas ex Hook.) Greene	Ogburn 287 (BRU)	KJ161554	-	-	-	-	KJ161726	KJ161784
Claytonia sibirica (L.) Howell	Ogburn n.a. (BRU) - Brown greenhouse	KJ161555, KJ161556	-	-	-	-	-	-
Montiopsis gayana (Barnéoud) D.I. Ford	Ogburn 268 (BRU)	-	-	-	-	-	KJ161727	KJ161785
Montiopsis gilliesii (Hook. & Arn.) D.I. Ford	Ogburn 166 (BRU)	KJ161557	-	-	-	-	-	KJ161786
Phemeranthus napiformis (DC.) Raf.	Ogburn 226 (BRU)	-	-	KJ161593	-	-	-	-
**Halophytaceae**								
Halophytum ameghinoi (Speg.) Speg.	Edwards 255 (BRU)	-	KJ161569	KJ161594	-	-	KJ161728	KJ161787
**Basellaceae**								
Anredera baselloides (Kunth) Baill.	Ogburn 256 (BRU)	KJ161558	KJ161570	KJ161595	-	-	-	KJ161788
Anredera cordifolia (Ten.) Steenis	913635/b (ZSS) - Hofstätter s/n, Madagascar	-	-	KJ161596	-	-	KJ161729	KJ161789
Anredera ramosa (Moq.) Eliasson	981287/0 (ZSS) - Mostul 1254, Mexico, Veracruz	-	-	KJ161597	-	-	-	-
Basella alba L.	Endara 1638 (FLAS) - Kanapaha Bot. Garden, Gainesville, FL	KJ161559	-	-	-	-	-	KJ161790
Ullucus tuberosus Caldas	101123/0 (ZSS) - Bot. Garden Bonn	-	-	KJ161598	-	-	KJ161730	KJ161791
**Didiereaceae**								
Alluaudia ascendens (Drake) Drake	823558/0 (ZSS) - received from Hauenstein, cultivated	KJ161560	-	-	KJ161625	-	-	-
Alluaudia procera (Drake) Drake	995323/0 (ZSS) - cultivated	KJ161561	-	KJ161599	KJ161626	KJ161642, KJ161643	KJ161731	KJ161792
Alluaudiopsis marnieriana Rauh	812213/0 (ZSS) - Bot. Garden Heidelberg	KJ161562	-	KJ161600	-	-	KJ161732	KJ161793
Ceraria longipedunculata Merxm. & Podlech	901396/0 (ZSS) - Lavranos & Bleck s/n, Namibia	KJ161563	-	KJ161601	-	KJ161644	-	KJ161794
Ceraria pygmaea (Pillans) G.D.Rowley	Lavranos 22309 (ZSS)	-	-	-	-	-	-	KJ161795
Decarya madagascariensis Choux	Rauh 7592 (ZSS)	-	-	-	-	-	KJ161733	KJ161796
Didierea madagascariensis Baill.	931246/0 (ZSS) - Rauh M1803, Madagascar	-	-	KJ161602	-	KJ161645, KJ161646	KJ161734	KJ161797
Portulacaria afra Jacq.	995326/0 (ZSS) - cultivated	-	KJ161571	KJ161603, KJ161604	-	-	KJ161735	KJ161798
**Talinaceae**								
Talinum arnotii Hook. f.	932096 (ZSS)	-	-	-	-	-	KJ161736	KJ161799
Talinum aurantiacum Engelm.	Ogburn 267 (BRU)	-	-	-	-	KJ161647	-	-
Talinum paniculatum (Jacq.) Gaertn.	941354/0 (ZSS) - Leuenberger & Eggli 4364, Argentina, Catamarca	-	KJ161572	KJ161605	-	KJ161648	KJ161737	KJ161800
Talinum sp.	961663/a (ZSS)	-	-	-	-	-	KJ161738	KJ161802
Talinum triangulare (Jacq.) Willd.	Ogburn n.a. (BRU) - Brown greenhouse	-	-	KJ161606	-	-	-	-
Talinella pachypoda Eggli	952546/0 (ZSS) - Hoffman 347, Madagascar	-	-	KJ161607	-	KJ161649	-	KJ161801
**Anacampserotaceae**								
Anacampseros albissima Marloth	Ogburn 255 (BRU)	-	KJ161573	KJ161608	KJ161627	-	KJ161739	KJ161803
Anacampseros filamentosa Sims	Ogburn 36 (MO)	-	KJ161574	-	KJ161628	-	-	-
Anacampseros kurtzii Bacigalupo	Spriggs n.a. (BRU) - Brown greenhouse	KJ161564	-	KJ161609	KJ161629	-	-	KJ161804

Anacampseros retusa Poelln.	803518/a (ZSS) - Cape Province	KJ161565	KJ161575	KJ161610	KJ161630	-	KJ161740	KJ161805
Grahamia bracteata Gillies ex Hook.	Ogburn n.a. (BRU) - Brown greenhouse	KJ161566	KJ161576	KJ161611	KJ161631	-	KJ161741	KJ161806
**Portulacaceae**								
Portulaca fulgens Griseb.	995813/0 (ZSS) - Leuenberger & Eggli 4896, Argentina, Tucuman	-	KJ161577	-	-	-	KJ161742	KJ161807
Portulaca grandiflora Hook.	998914/0 (ZSS) - Hunkeler 591, Argentina, Catamarca	-	KJ161578, KJ161579	-	-	-	-	-
Portulaca molokiniensis Hobdy	921793/0 (ZSS) - Wong n.a., Hawaii, Molokini Island	-	-	-	-	-	KJ161743	KJ161808
Portulaca oleracea L.	Sage 0509-01	-	KJ161580	-	-	-	KJ161744, KJ161745	KJ161809
**Cactaceae - Pereskia**								
Pereskia aculeata Mill.	Ogburn n.a. (BRU) - Brown greenhouse	X95860, KJ161567	-	KJ161612	KJ161632	-	-	-
Pereskia aureiflora F. Ritter	166-54-83-20 (B) - R. Nyffeler	KJ161568	-	KJ161613	KJ161633	KJ161650	KJ161746	KJ161810
Pereskia bleo (Kunth) DC.	Ogburn n.a. (BRU) - Brown greenhouse	-	-	KJ161614	-	KJ161651	KJ161747	KJ161811
Pereskia diaz-romeroana Cárdenas	Arakaki n.a. (BRU) - Brown greenhouse	-	-	KJ161615	KJ161634	KJ161652	KJ161748	KJ161812
Pereskia grandifolia Haw.	Ogburn n.a. (BRU) - Brown greenhouse	-	-	KJ161616	KJ161635	KJ161653	KJ161749	KJ161813
Pereskia guamacho F.A.C. Weber	Arakaki n.a. (BRU) - Brown greenhouse	-	-	KJ161617	KJ161636	-	KJ161750	KJ161814
Pereskia portulacifolia (L.) DC.	Ogburn n.a. (BRU) - Brown greenhouse	-	-	KJ161618	KJ161637	KJ161654	KJ161751	KJ161815
Pereskia quisqueyana Alain	Ogburn n.a. (BRU) - Brown greenhouse	-	-	-	KJ161638	-	-	KJ161816
Pereskia sacharosa Griseb.	Arakaki n.a. (BRU) - Brown greenhouse	-	-	-	-	-	KJ161752	KJ161817
Pereskia zinniiflora DC.	842526/a (ZSS) - Cuba, Sancti Spiritus	-	KJ161581	-	KJ161639	-	KJ161753	KJ161818
**Cactaceae - Opuntioideae**								
Brasiliopuntia brasiliensis (Willd.) A. Berger	Ogburn n.a. (BRU) - Brown greenhouse	-	-	-	-	KJ161655, KJ161656	-	-
Cylindropuntia kleiniae (DC.) F.M. Knuth aff	Ogburn n.a. (BRU) - Brown greenhouse	-	KJ161582	-	-	KJ161657, KJ161658	KJ161754	KJ161819
Maihueniopsis glomerata (Haw.) R. Kiesling	Arakaki 1905 (BRU) - 55 from Mesa Garden Nursery 1.932 (D. Ferguson 280), Argentina, Salta	-	KJ161583	-	-	-	KJ161755	KJ161820
Nopalea cochenellifera (DC.) F.M. Knuth aff	Ogburn n.a. (BRU) - Brown greenhouse	-	-	KJ161830	-	KJ161659, KJ161660	KJ161756	KJ161821
Pereskiopsis diguetii (F.A.C. Weber) Britton & Rose	942160/b (ZSS) - Lomelí Sención & Díaz L. s/n, Mexico, Jalisco	-	KJ161584	-	-	KJ161661	KJ161757	KJ161822
Pereskiopsis gatesii Baxter	Ogburn n.a. (BRU) - Brown greenhouse	-	KJ161585	KJ161619	-	KJ161662, KJ161663	-	-
Pereskiopsis porteri (Brandegee ex F.A.C. Weber) Britton & Rose	Ogburn n.a. (BRU) - Brown greenhouse	-	-	-	-	KJ161664, KJ161665	KJ161758	KJ161823
Quiabentia verticillata (Vaupel) Vaupel ex Berger	Ogburn n.a. (BRU) - Brown greenhouse	-	KJ161586	-	-	KJ161666	-	-
Quiabentia zehntneri (Britton & Rose) Britton & Rose	864369 (ZSS)	-	-	-	-	-	KJ161759	KJ161824
Tephrocactus articulatus (Pfeiff.) Backeb.	Spriggs n.a. (BRU) - Brown greenhouse	-	KJ161587	KJ161620	-	KJ161667	KJ161760	KJ161825
**Cactaceae - Cactoideae**								
Acanthocereus tetragonus (L.) Hummelinck	892219 (ZSS) - brought by R. Nyffeler	-	-	-	-	KJ161668, KJ161669	-	-
Coryphantha radians (DC.) Britton & Rose	Arakaki 1898 (BRU) - from Mesa Garden Nursery 125 (S.Brack 679), Mexico, Hidalgo	-	KJ161588	-	-	KJ161670	-	KJ161826

Echinocactus grusonii Hildm.	Spriggs n.a. (BRU) - Brown greenhouse	-	-	KJ161621	KJ161640	-	-	-
Echinocereus pectinatus (Scheidw.) Engelm.	-	-	-	KJ161831	-	-	-	-
Hylocereus undatus (Haw.) Britton & Rose	-	-	-	JF966382	-	JF966381	-	-
Mammillaria plumosa F.A.C. Weber	Arakaki 1907 - from Mesa Garden Nursery 845.5 (S. Brack 327), Mexico, Coahuila	-	KJ161589	KJ161622	-	KJ161671	-	KJ161827
Selenicereus wittii (K. Schum.) G.D. Rowley	-	-	-	-	-	-	-	-
Thelocactus leucacanthus (Zucc. Ex Pfeiff.) Britton & Rose	Arakaki 1912 (BRU) - from Mesa Garden Nursery 1273 (S. Brack 514), Mexico, Hidalgo	-	KJ161590	KJ161623	KJ161641	KJ161672, KJ161673	KJ161761	KJ161828
Weingartia kargliana Rausch	782218/b (ZSS) - Rausch 677, Bolivia, N of Tupiza	-	-	KJ161624	-	KJ161674	-	KJ161829

Supplementary Table 3: Additional primers used for PCR amplification of Caryophyllales genes encoding PEPC.

Name	Primer	Target
ppc-1588-For	GGKGCATACATYATCTCCATG	all *ppc-1E1*
ppc-1621-For	TCRGATGTGCTTGCTGTTGAG	all *ppc-1E1*
ppc-1792-For	ATGATTGGRTACTCTGATTC	all *ppc-1E1*
ppc-2261-For	TGAACATTGGAAGCCGTCC	all *ppc-1E1*
ppc-2761-Rev	CTTGGATAAGTGAGGCCTCTC	*ppc-1E1a*
ppc-2831-Rev	CAGGAGCATACTCACTGGAGG	*ppc-1E1b*
ppc-2846-Rev	GGTGTCCTCAAGGCCAGGAG	all *ppc-1E1*
ppc-2874-Rev	GCAGCAATACCTTTCATGGTG	*ppc-1E1c*
ppc-2748-Rev	ATGCCATYAGTTCTTTGGATAAG	*ppc-1E1c*
ppc-1363-For	ATTCGYCAAGAATCTGACCG	*ppc-1E2*
ppc-2452-Rev	GTRACCCTAAAGAAAGGCCATTC	*ppc-1E2*
ppc-1884_For	GCARGGAGARATGGTRCAGGC	*ppc-2*
ppc-2633-Rev	ATRGTGATSAGCAATGCRTCTC	*ppc-2*
ppc-2651-Rev	AGCAGCAATYCCATTAATAG	*ppc-2*

Supplementary Table 4: Water treatment of *Portulaca oleracea* plants

Date	Group A	Group B
05.03.2012	watered[1]	watered[1]
08.03.2012	watered	
11.03.2012	watered	
13.03.2012	watered	
15.03.2012	watered	watered
17.03.2012	watered	
19.03.2012	watered	
22.03.2012	watered	watered
26.03.2012	watered	
31.03.2012	watered[1]	watered[1]
04.04.2012	watered	
06.04.2012	sampled	sampled

[1]Nutrients added

Supplementary Table 5: Sequencing and mapping statistics.

Time	Day		Night		Day		Night	
Condition	Watered				Drought			
Individual	1	2	1	2	3	4	3	4
Pairs of reads sequenced	11,980,617	7,440,608	7,519,679	11,832,946	7,311,777	8,168,471	8,659,554	7,785,604
Pairs of reads used in assemblies	10,017,935	5,886,163	5,708,027	6,352,153	6,286,010	6,352,153	7,403,534	6,957,525
Contigs	47,047	44,957	43,749	66,755	40,334	56,987	48,471	55,248
Percent reads aligned	89.11	86.84	86.98	80.58	88.3	86.96	87.45	86.34

Supplementary Table 6: Expression levels in rpm of C_4-related genes in day and night samples of *Portulaca oleracea* plants grown in different conditions[1].

Time	Day		Night		Day		Night	
Condition	Watered				Drought			
Individual	1	2	1	2	3	4	3	4
ALA-AT-1	2,382	1,095	3,723	2,722	775	306	651	539
ASP-AT-1	83	105	175	134	78	202	83	96
ASP-AT-2	743	500	444	222	438	162	120	90
ASP-AT-3	0	0	0	0	0	0	0	0
ASP-AT-4	2,111	1,430	1,141	798	680	650	329	375
β-CA-1	0	0	0	0	0	0	0	0
β-CA-2	714	649	273	298	332	423	255	253
β-CA-3	658	325	291	139	208	66	53	28
β-CA-4	20,880	10,296	9,775	6,476	10,818	2,021	1,631	1,505
NAD-MDH-1	22	12	15	19	16	74	40	35
NAD-MDH-2	675	532	363	197	630	628	207	179
NAD-MDH-3	1171	1168	292	239	837	371	146	132
NAD-ME-1	643	666	534	501	648	499	535	560
NAD-ME-2	1131	990	947	804	838	617	636	591
NADP-MDH-1	1,956	2,300	20	2,359	1,357	1,793	1,812	2,263
NADP-MDH-2	13	231	40	11	4	21	10	20
NADP-MDH-3	648	520	534	523	939	764	1,141	1,246
NADP-ME-1	1,184	896	215	313	1,021	651	385	281
NADP-ME-2	0	0	0	17	0	0	0	0
NADP-ME-3	42	19	102	0	75	86	211	257
PCK-1	21	13	28	77	10	11	41	48
PPDK-1	26,696	24,814	21,010	21,105	29,855	18,778	11,463	10,164

[1] ALA-AT = alanine aminotransferase, ASP-AT = aspartate aminotransferase, β-CA = β-carbonic anhydrase, NAD-MDH = NAD-malate dehydrogenase, NAD-ME = NAD-malic enzyme, NADP-MDH = NADP-malate dehydrogenase, NADP-ME = NADP-malic enzyme, PCK = phosphoenolpyruvate carboxykinase, PPDK = pyruvate, phosphate dikinase.
